# Anti-tumour necrosis factor therapy for early-stage Dupuytren’s disease (RIDD): a phase 2b, randomised, double-blind, placebo-controlled trial

**DOI:** 10.1016/S2665-9913(22)00093-5

**Published:** 2022-04-29

**Authors:** Jagdeep Nanchahal, Catherine Ball, Ines Rombach, Lynn Williams, Nicola Kenealy, Helen Dakin, Heather O’Connor, Dominique Davidson, Paul Werker, Susan J Dutton, Marc Feldmann, Sarah E Lamb

**Affiliations:** Kennedy Institute of Rheumatology, University of Oxford, Oxford, UK; Kennedy Institute of Rheumatology, University of Oxford, Oxford, UK; Centre for Statistics in Medicine, Oxford Clinical Trials Research Unit, Nuffield Department of Orthopaedics, Rheumatology and Musculoskeletal Sciences, Health Economics Research Centre University of Oxford, Oxford, UK; Kennedy Institute of Rheumatology, University of Oxford, Oxford, UK; Kennedy Institute of Rheumatology University of Oxford, Oxford, UK; Health Economics Research Centre University of Oxford, Oxford, UK; Centre for Statistics in Medicine, Oxford Clinical Trials Research Unit, Nuffield Department of Orthopaedics, Rheumatology and Musculoskeletal Sciences University of Oxford, Oxford, UK; Department of Plastic Surgery, St John’s Hospital, Livingston, Scotland, UK; Department of Plastic Surgery, University Medical Center Groningen and University of Groningen, Groningen, The Netherlands; Centre for Statistics in Medicine, Oxford Clinical Trials Research Unit, Nuffield Department of Orthopaedics, Rheumatology and Musculoskeletal Sciences University of Oxford, Oxford, UK; Kennedy Institute of Rheumatology University of Oxford, Oxford, UK; College of Medicine and Health, University of Exeter, St Luke’s Campus, Exeter, UK

## Abstract

**Background:**

Dupuytren’s disease is a common fibrotic condition that causes the fingers to flex irreversibly into the palm. Treatments for late-stage disease all have limitations, and there is no approved treatment for early-stage disease. We identified tumour necrosis factor as a therapeutic target in Dupuytren’s disease, and in a dose ranging trial found 40 mg adalimumab in 0·4 mL to be most efficacious. Here we aimed to assess the effects of intranodular injection of adalimumab in early-stage disease.

**Methods:**

In this phase 2b, randomised, double-blind, placebo-controlled trial adults with early-stage Dupuytren’s disease and an established clinically distinct nodule with a clear history of progression in the preceding 6 months were recruited from two clinical centres in the UK and were randomly assigned 1:1 to receive four injections of adalimumab or saline every 3 months. Participants and assessors were masked. The primary outcome was nodule hardness measured with a durometer at 12 months. Data were analysed by linear mixed effects regression models in the intention-to-treat population with multiple imputation for missing primary outcome data. The trial is registered at the ISRCTN registry, ISRCTN 27786905 and is complete.

**Findings:**

Between Feb 17, 2017, and Jan 11, 2019, 284 participants were screened in the UK and 140 were enrolled. 47 (34%) participants were female and 93 (66%) were male. Mean age of participants was 59·7 years (SD 10·0). Primary outcome data were available from 113 participants. Nodule hardness was lower (−4·6 AU [95% CI −7·1 to −2·2], p=0·0002) in the adalimumab compared with the saline group at 12 months. There were no related serious adverse events; the most common adverse events were minor injection site reactions.

**Interpretation:**

Intranodular injections of adalimumab in participants with early-stage Dupuytren’s disease resulted in softening and reduction in size of the nodules. Longer follow-up would be required to assess the effect of tumour necrosis factor inhibition on disease progression, extension deficit and hand function.

## Introduction

Dupuytren’s disease is a common fibrotic condition involving the palm of the hand and affects around 4−7% of the general population in the UK and USA. It is more prevalent in older people, with 12% of those aged 55 years and 29% of those aged 75 years being affected in western countries.^1^ There is a strong genetic component, with heritability estimated at 80%.^2^ The early stages of the disease manifest as nodules; progression to fibrous cords occurred in about 20% of individuals over the course of 7 years in one study;^3^ in another study, about 35% of individuals progressed over the course of 18 years.^4^ Patients with Dupuytren’s diathesis are considered to have more aggressive disease progression. Factors associated with diathesis include early age of onset (<50 years), positive family history, and ectopic disease.^5^ The cords cause flexion deformities of the fingers, which can significantly affect activities of daily living. Existing guidance is for treatment to be deferred until the finger joints are flexed to 30° with impairment of hand function,^6^ when the diseased tissue can be surgically excised, or the cords can be disrupted by means of a needle or collagenase. However, the disease recurs in 21% of patients following surgical excision (fasciectomy) and in 85% following needle fasciotomy at 5 years.^7^ Treatments for late-stage disease are also associated with potential complications, ranging from transient swelling and bruising to nerve and tendon injury.^8,9^

The ideal treatment would be effective during the early nodular stages of the disease to prevent the development of cords and progression to finger contractures. Surgical excision of early-stage disease is associated with high risk of recurrence.^10^ Our systematic review^11^ identified various non-surgical treatments used for patients with early-stage disease. Intranodular steroid injections and radiotherapy have been reported to lead to subjective softening of the nodules and retard disease progression. However, all the studies identified in that systematic review were poorly designed, with no controls, and neither the observers nor patients were masked.^11^ A randomised, double-blind, placebo controlled trial of collagenase injection reported reduction in nodule size and hardness over an 8-week period.^12^ More recently, a randomised trial of 52 patients reported that extracorporeal shock wave therapy reduced pain.^13^

We have shown that the nodules of patients with early-stage Dupuytren’s disease are a complex ecosystem of myofibroblasts and fibroblasts, each comprising several subpopulations, including highly contractile myofibroblasts, a separate cycling myofibroblast population, and immune regulatory fibroblasts,^14^ together with a minority (11%) of immune cells.^15^ Dupuytren’s disease is a localised inflammatory disorder, with differentiation and activation of myofibroblasts promoted by TNF expressed by local M2 macrophages and mast cells acting via TNF receptor 2 and the canonical Wnt signalling pathway.^15,16^ Genome-wide association studies have also highlighted the role of Wnt signalling in Dupuytren’s disease.^17^ In a dose ranging phase 2a study, we found that intranodular injection of 40 mg adalimumab in 0·4 mL resulted in down-regulation of the myofibroblast phenotype as characterised by reduced expression of alpha-smooth muscle actin (α-SMA) and procollagen type I proteins 2 weeks after injection.^18^ Here, we report the outcome of a randomised, phase 2b trial comparing four injections of adulimumab or placebo given once every 3 months in participants with early-stage Dupuytren’s disease.

## Methods

### Study design and participants

Repurposing anti-TNF for Dupuytren’s disease (RIDD) is a phase 2, randomised, participant and assessor masked (double-blinded) placebo-controlled study to assess the efficacy of local injection of adalimumab in participants with early-stage Dupuytren’s disease. The protocol was approved by the South Central Oxford B Research Ethics Committee (reference number 15/SC/0259) and the Medicine and Healthcare products Regulatory Authority (EudraCT no 2015-001780-40), and has been published.^19^ Protocol amendments were mostlyminor (appendix pp 2−5). Our original intention was to recruit from a total of three centres in the UK and use a standard durometer. We were unable to open the third centre in the UK. Therefore, we opened the study in the Netherlands, where we used the slim probe durometer (Rex Gauge RX-1600-OO) because we anticipated a more aggressive disease phenotype in these patients (appendix pp 6−7). Our intention was to combine the standard and slim durometer measurements from the UK and Dutch populations by means of a cross-walk model. However, this was not possible (appendix p 8) and the data from the Dutch cohort are presented separately (appendix pp 6−12). The original target sample size was achieved for the UK cohort.

This study was done as part of the portfolio of trials in the registered UKCRC Oxford Clinical Trials Research Unit at the University of Oxford. It has followed their Standard Operating Procedures ensuring compliance with the principles of Good Clinical Practice and the Declaration of Helsinki and any applicable regulatory requirements. Two UK centres (Oxford University Hospitals NHS Trust, Oxford and Wellcome Trust Clinical Research Facility, Edinburgh) recruited adult participants older than 18 years through posters displayed in general practitioner surgeries and information on the British Dupuytren’s Society website, the trial website, and Facebook and Twitter accounts. Eligibility criteria included adults with early-stage Dupuytren’s disease (active extensor deficit at the metacarpophalangeal or the interphalangeal joints of the affected ray of ≤30°) and an established clinically distinct nodule with a clear history of progression in the preceding 6 months. Criteria for progression included patient-reported increase in nodule size, pain or tenderness, and itching. We recruited participants with no previous treatment for Dupuytren’s disease to the affected digit or radiotherapy to the hand and screened them for tuberculosis, HIV, and hepatitis B and C using serological testing and chest x-ray in accordance with local standard procedures for anti-TNF screening. Participants with significant renal, hepatic, or systemic inflammatory disease, moderate or severe heart failure, demyelinating disorder, history of repeated infections, treatment with coumarin anticoagulants, concomitant disease modifying anti-rheumatic drugs treatment, or history of cancer were excluded. All participants provided written, informed consent before enrolment.

### Randomisation and masking

Participants were randomly assigned 1:1 by means of variable permuted blocks and stratification for trial centre and age (18−49 *vs* ≥50 years). The randomisation was computer generated once each participant was registered onto the trial by means of a telephone-web-based system administered independently by the Oxford Clinical Trials Research Unit.^19^ The adalimumab or saline were drawn up in a separate room and the syringe and needle used for injection were identical, maintaining masking of the participant. The medically qualified health professional doing the injection did not assess any of the outcomes, which were done by a masked individual who was not present during the preparation and delivery of the injection.

### Procedures

The most active nodule as reported by the participant was selected for inclusion in the study and participants were randomly assigned 1:1 to receive either 40 mg adalimumab in 0·4 mL or an equal volume of saline at baseline, 3, 6, and 9 months after randomisation, and followed up at 12 and 18 month timepoints. The 40 mg in 0·4 mL preparation of adalimumab is only available in a prefilled glass syringe fitted with a non-removable 29-gauge needle. We had previously found that it was not possible to inject into the dense substance of the nodule without applying undue pressure on the plunger, which was associated with increased pain. Therefore, following guidance from the pharmacy at Oxford University Hospitals, all centres transferred the drug into a 1 mL disposable syringe just before use and injected using a 1 inch long 25-gauge needle. Nodule hardness was measured by means of a durometer (Rex Gauge RX-1600-OO standard probe, Rex Gauge Company, IL, USA). Nodule size was assessed by means of ultrasound scan (GE Logiq E R6 with L4-12t-RS probe [depth 2· 5 cm, 12 MHz] GE Healthcare, Wauwatosa, WI, USA). The nodule was marked and photographed with a scale marker, active and passive extension deficit of the joints of the affected digit was measured by means of a goniometer (Promedics, Port Glasgow, UK), and grip strength was assessed by means of a Jamar hand meter Sammons Preston, Bolingbrook, IL USA). The study nodule was injected after completion of all the measurements, and pain scores were recorded during and immediately after the injection, as well as any adverse effects at the injection site.

We recorded all systemic adverse events graded 3 or above in accordance with the protocol. In view of the well documented safety profile of adalimumab, the frequency of injection (once every 3 months as opposed to the usual twice a week for inflammatory arthritis) and the limited number of injections (total of four), we and the regulators did not feel that collection of adverse events below grade 3 was necessary. We recorded all local adverse events, irrespective of severity.

### Outcomes

We assessed nodule hardness as the primary outcome measure as it has been used in previous studies examining the effect of intralesional steroid injection, radiotherapy,^11^ or collagenase.^12^ We used a standard probe durometer to quantitatively assess nodule hardness at 12 months as our primary outcome, with data for this and most secondary outcomes also being collected at 3, 6, 9, 12, and 18 months. The measurement site was marked on the skin at the baseline visit and photographed with a scale marker. This was used as a reference for measurement on each subsequent occasion to ensure consistency. Secondary outcomes included ultrasound-based measurement of the nodule cross-sectional area in the sagittal plane, nodule height, and maximum feret (maximum diameter between two parallel lines in any plane by means of ImageJ, National Institutes of Health, Bethesda, MD, USA), by a single masked observer (CB). The underlying tissue landmarks, including the skeleton, were used to ensure that the same cross-sectional site was imaged on each occasion. Other secondary outcomes comprised extension deficit of the joints of the affected digit, grip strength, patient reported outcome measures^20^ (Michigan Hand Questionnaire [MHQ] and most restricted activity), progression to surgery, injection experience (pain during and immediately after injection), and adverse events. EuroQol five-dimensional five level instrument and resource use were measured and are reported separately in a parallel health economic analysis, which will be detailed in a follow-up report.

To assess whether the development of antibodies to adalimumab correlated with outcomes, tertiary outcomes at 3 months and 12 months included concentrations of antibodies to adalimumab. These were initially established by means of a semiquantitative screening assay (IDK monitor ADA assay, Immundiagnostik, Bensheim, Germany) followed by a quantitative assay (RIDA anti-ADM, R-Biopharm, Darmstadt, Germany) for those that exceeded the screening assay threshold. We also measured concentrations of circulating adalimumab (IDKmonitor assay, Immundiagnostik, Bensheim, Germany) at 3 months and 12 months.

### Statistical analyses

Statistical analyses were done according to the protocol^19^ and statistical analysis plan, which was approved by the trial management team and finalised before the data were received for the final analysis (changes from those prespecified in appendix p 2). Raw data were accessed by IR and HO’C and the analyses were verified by an independent statistician at the Oxford Clinical Trials Research Unit. We estimated the number of participants required for the study by means of the standard durometer as the primary trial endpoint based on a preliminary pilot study on nodules in 25 participants with early stage Dupuytren’s disease compared with the same anatomical site on the palm of age and sex matched controls. In this pilot study, the mean durometer reading was 53 arbitrary units (AU; SD 8) compared with 32 AU (SD 3) at equivalent sites on the palm in healthy controls.^21^ On this basis, we estimated that a minimum of 138 participants (69 per treatment group) were required to achieve 90% power with a two-sided 5% significance level to detect a moderate standardised effect size of 0·625, and allowing for 20% loss of follow-up. Assuming a common SD of 8, the effect size of 0·625 corresponds to a five-point change in nodule hardness measured by the standard durometer.

Durometer readings were not available on some occasions (117 [14%] of 840), especially at later timepoints when in some participants progression of the disease precluded reliable assessment owing to the relatively wide base plate of the standard durometer. However, ultrasound scan measurements were available for these participants, and missing data for the primary endpoint were handled by multiple imputation by chained equations using predictive mean matching (which used the ten closest observations).^22^ The imputation model included durometer data, nodule area and feret, flexion deformity, randomisation site, randomisation date, and participant age. Missing baseline data were mean imputed before the imputation, which was run separately by treatment group. 50 imputations were generated. The primary outcome was analysed by means of linear mixed effects regression models adjusting for baseline values of the outcome variable and stratification factors (trial site and age). Secondary outcomes were analysed by means of similar statistical models, including all available data without imputation of missing follow-up data; missing baseline data were mean imputed. Treatment effects with corresponding 95% CI were estimated for each follow-up timepoint, and the 12-month estimates were considered the follow-up of principal interest. Sensitivity analyses accounted for the effect of missing data by considering missing not at random scenarios (ie, whether participants with missing data might have had outcomes substantially better or worse than those with available follow-up data), the effect of delayed assessments due to COVID-19, and the per-protocol population, excluding participants with fewer than three injections, or those who received a non-randomised treatment or surgery during the follow-up. Unmasked data were analysed by the trial statisticians (IR, HO’C, and SD) and subsequently made available to the remainder of the trial team. The trial was monitored by independent data monitoring and safety, and trial steering committees. The trial was registered on ISRCTN (27786905) and ClinicalTrials.gov (NCT03180957).

### Role of funding source

The study was funded by the Health Innovation Challenge Fund (Wellcome Trust and Department of Health). Funding for purchase of the adalimumab was provided by 180 Life Sciences Corp. The funders had no involvement in study design or data analyses. The study was sponsored by the University of Oxford.

## Results

**Figure 1:**
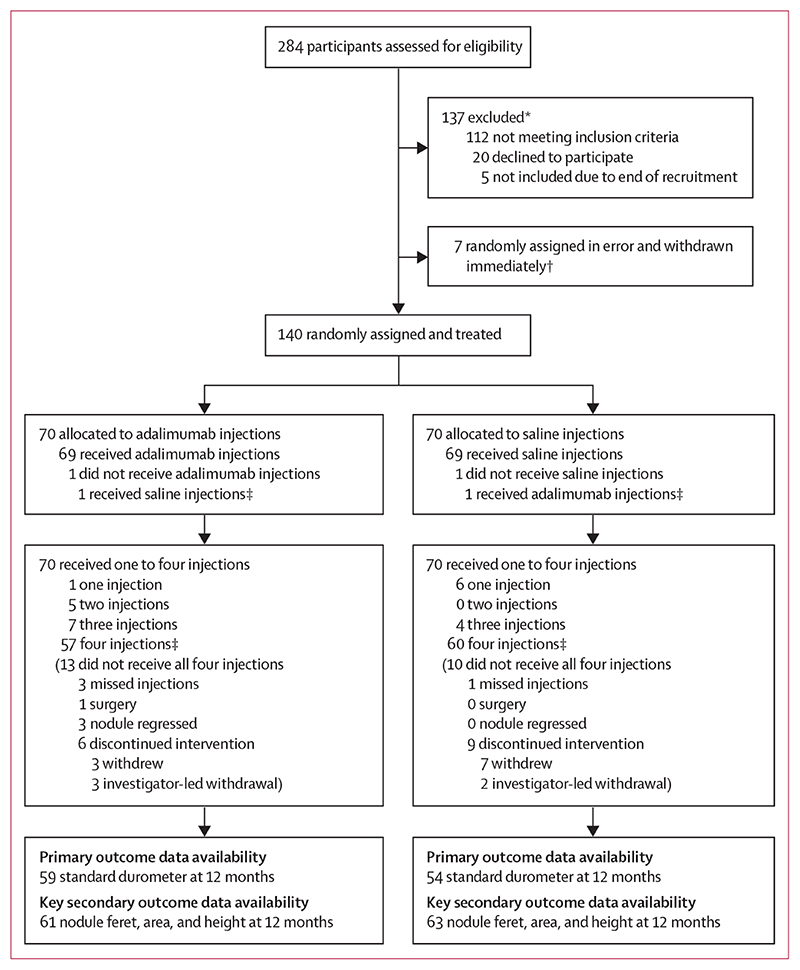
Trial profile *Includes participants excluded due to contraindications to adalimumab (tuberculosis n=7, HIV or hepatitis B n=1, systemic inflammatory disease n=3, and history of cancer n=26). ^†^Total of seven participants (three in the adalimumab group, four in the saline group) were randomly assigned erroneously before their baseline assessment and withdrawn immediately from all study involvement; they are not included in any of the subsequent summaries. ^‡^One participant randomly assigned to adalimumab received saline injections throughout the trial. One participant randomly assigned to saline injections received one adalimumab injection at month 3; both are included as having received their injections.

Between Feb 17, 2017, and Jan 11, 2019, 284 participants were screened in the UK and 140 were randomly assigned (figure 1). 47 (34%) participants were female and 93 (66%) were male. Mean age of participants was 59·7 years (SD 10·0). Overall, baseline characteristics were similar between the two groups (table 1).

**Figure 2:**
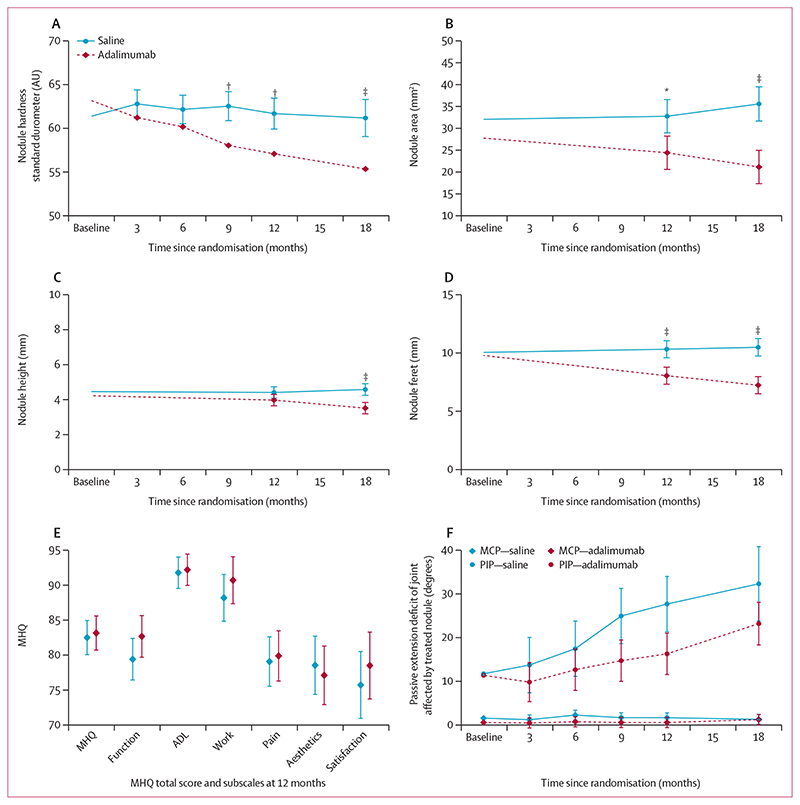
Changes in nodule hardness (standard durometer), area, height, and feret on ultrasound scan, MHQ, and passive range of motion of affected joint over time Change in nodule hardness (standard durometer; A), nodule area (B), nodule height (C), nodule feret (D), MHQ (E), and passive extension deficit for MCP or PIP joints affected by study nodule (F; number of affected joints at baseline MCP [adalimumab n=53, saline n=60], PIP [adalimumab n=16, saline n=10]. Data shown as point estimates of adjusted marginal means and 95% CIs. MHQ=Michigan Hand Questionnaire. ADL=activities of daily living. MCP=metacarpophalangeal. PIP=proximal interphalangeal. *p=0·0025. ^†^p=0·0002. ^‡^p<0·0001.

The primary outcome of nodule hardness at 12 months was significantly lower in the adalimumab group (mean adjusted marginal difference based on multiply imputed data −4·6 AU [95% CI −7·1 to −2·2], p=0· 0002; figure 2A, table 2). Supplemental and sensitivity analyses, including assessing the effect of missing data on the trial results, confirmed the primary results (appendix pp 13−15) and results were consistent when compared across important prognostic factors (appendix p 16). Nodule hardness was assessed at other timepoints as a secondary outcome and decreased further at 18 months (mean adjusted difference −5·8 AU [−8·7 to −3·0], p<0·0001; figure 2A and table 2).

Nodule area was significantly lower in the adalimumab group at 12 months (mean adjusted difference −8·4 mm^2^ [95% CI −13·8 to -2·9], p=0·0025) and decreased further by 18 months (mean adjusted difference −14·4 mm2 [−19·9 to −9·0], p<0·0001; figure 2B, table 2, and appendix p 17). The adalimumab and saline groups did not differ significantly in nodule height at 12 months (mean adjusted difference −0·4 mm [−0·9 to 0·0], p=0·064), although there was a significant difference at 18 months (mean adjusted difference −1·1 mm [−1·5 to −0·6], p<0· 0001; figure 2C, table 2, and appendix p 17). Maximum nodule feret was significantly lower in the adalimumab compared with the saline group at 12 months (mean adjusted difference −2·3 mm [−3·3 to −1·2], p<0·0001, and decreased further at 18 months (mean adjusted difference −3·3 mm [−4·3 to −2·2], p<0·0001; figure 2D, table 2, and appendix p 17).

The scores for the MHQ were similar for the adalimumab and saline-treated groups (figure 2E; appendix pp 18−20). The joint adjacent to the treated nodule was considered to be the affected joint as assessed by a masked observer from baseline photographs.^23^ The passive extension deficit for the metacarpophalangeal joints affected by the treated nodules was also similar between the two groups, and although it appeared to be better for the adalimumab-treated participants when the nodules affected the proximal interphalangeal joint, the number of participants with involvement of this joint were too low (16 participants in the adalimumab group, ten participants in the placebo group) to draw any meaningful comparisons (figure 2F, appendix pp 22−23). The most restricted activity, grip strength and active extension deficit for the joints affected by the treatednodules were similar for the two treatment groups (appendix pp 20−22). During injection, the median pain score was 8 in the adalimumab group, and 7 in the saline group (on a 1−10 scale) and decreased in both groups to a median of 2 immediately after injection (appendix p 24). On approximately one-third of occasions, participants chose application of a topical local anaesthetic cream (EMLA or Ametop) before injection. This did not affect the overall pain scores. By the 18-month follow-up, ten participants in the saline group had undergone or were awaiting surgery, all related to the study nodule. In the adalimumab group, three underwent surgery during the course of the trial (all unrelated to the study nodule) and five were awaiting surgery at 18 months (three related to the study nodule). These participants who underwent surgery during the course of the trial, or who were awaiting surgery at the end of the follow-up period, were not treated by surgeons involved in the trial.

The majority (128 [91%] of 140) participants received at least three of the four injections offered to them and 117 (84%) of 140 received all four injections. Circulating concentrations of adalimumab at 3 months and 12 months were negligible in the saline group and low in the adalimumab group (0·0 [0·0 to 0·6] μg/mL; median [IQR]; appendix p 25). At 12 months, by means of a quantitative assay, 48 (80%) of 60 of the participants randomly assigned and treated with adalimumab had antibodies to adalimumab higher than the threshold for detection, with a median (IQR) concentration of 29·8 ng/mL (2·4 to 128·8; appendix p 26). There was no relationship between the concentration of circulating antibodies to adalimumab and change in durometer readings or nodule area, feret, or height at 12 months compared with baseline (appendix pp 27−29).

One participant in the placebo group developed pericarditis, which was considered unrelated to the saline injection. No serious adverse events related to treatment were recorded. Local adverse events were minor injection site reactions (itching, redness, bruising, haematoma) and were recorded on 25 occasions (saline=16, adalimumab=nine; table 3). There were no nerve injuries.

## Discussion

Our results show that intranodular injections of 40 mg adalimumab in 0·4 mL are effective in reducing nodule hardness and nodule size. This builds on our previous phase 2a dose-ranging study, where we found that this preparation of adalimumab downregulated the myo-fibroblast phenotype as evidenced by reduced concentrations of α-SMA and procollagen type I proteins.^18^

Adalimumab is usually administered every 2 weeks for systemic inflammatory disorders. Our decision to administer four doses at 3 month intervals was based on the results of an end user survey of 40 participants, 20 with early-stage and 20 with late-stage Dupuytren’s disease, regarding the number of injections they would find acceptable per annum. Adalimumab has a mean half-life of 2 weeks. Our findings that the nodules continued to soften and regress on ultrasound at the 18-month timepoint, 9 months after administration of the final injection, suggests that local administration has a profound local biological effect. We have previously shown that Dupuytren’s disease is a low-grade localised inflammatory disorder and the myofibroblast phenotype characterised by expression of α-SMA and collagen type I, and contractility is critically dependent on the production of low concentrations of TNF locally.^15,16^ It is possible that the intermittent local administration of a relatively high dose of adalimumab could be sufficient to significantly affect the relatively small pool of cycling myofibroblasts^14^ or promote myofibroblast apoptosis. Only nodules showing signs of clinical activity and progression (increase in size, pain or tenderness, and itching) would need to be treated, and we anticipate that each of these nodules would need to be injected separately. We would envisage that following completion of a course of four injections, nodule development could be followed expectantly, and the treatment repeated in the event of reactivation of the disease. We have previously shown that plasma concentrations of adalimumab 2 weeks following injection into Dupuytren’s nodules are similar to when it is administered systemically in patients with rheumatoid or psoriatic arthritis.^18^ It is possible that effects might be more pronounced and extend over a more prolonged time with a depot preparation of adalimumab, which currently does not exist.

When adalimumab is administered systemically, development of antidrug antibodies is associated with poorer disease control.^24^ The relatively high concentrations of circulating anti-adalimumab antibodies seen in some of our participants might be related to lack of administration of concomitant disease modifying agents such as methotrexate, which are usually administered in patients with rheumatoid arthritis.^25^ However, we did not find a correlation between the concentration of anti-adalimumab antibodies and response (nodule hardness or nodule area, height, maximum feret). This might be owing to the high dose delivered locally not being influenced by subsequent clearance from the circulation.

Palmar injections are painful owing to the high density of innervation of the skin and large cortical representation, resulting in median visual analogue pain scores of around 7−8/10 during the injection. However, the pain rapidly subsided to a median of 2/10 immediately after the injection. As reported previously,^18^ there was no difference in pain scores between the citrate-free concentrated formulation of adalimumab (excipients mannitol, polysorbate, and water) and saline.

There is a lack of consensus on the optimal outcome measures for patients with late-stage Dupuytren’s disease^20^ and even more so for patients with early-stage disease. Previous studies assessing the effect of intranodular steroid injections or radiotherapy have relied on subjective assessment of nodule hardness and size.^11,20^ The Rex Gauge Type OO standard durometer was previously been found to be reliable and sensitive for assessing skin hardness in patients with scleroderma.^26^ Before the report of the use of a durometer in early-stage Dupuytren’s disease,^12^ we trialled the standard durometer in a small pilot study^21^ before using it to quantitatively assess nodule hardness. The slim durometer, which has a smaller baseplate that is less likely to impinge on the surrounding skin, was used in participants from the Netherlands as the anticipated accelerated disease progression would have made it more challenging to use the standard probe at later timepoints. We had expected that the readings for the standard and slim durometers would be similar as they are identical (Rex Gauge RX-1600-OO) except for the diameter of the foot plate. However, our data show that the slim durometer is less accurate, and we were unable to use crosswalk methodology to map the results from the standard durometer to those from the slim durometer. We achieved the sample size in the UK that was consistent with our original protocol specification. Therefore, our main analysis used outcomes for the standard durometer in the UK population and we would recommend it be used in future studies.

Previous studies also relied on subjective assessment of nodule size.^11,20^ Measurement of nodule surface area has been reported to be subject to relatively low intraobserver and interobserver agreement for some digits.^27^ We used ultrasound imaging to assess nodule size more reliably. A systematic review^28^ concluded that ultrasound scans would be useful for following the course of early-stage disease, a finding subsequently validated in a cohort of 50 patients.^29^ An operator masked to the treatment allocation did all the ultrasound scans in the UK and also did all of the measurements. Reliability of sequential imaging was improved by ensuring that the images were recorded with the same deep landmarks, including the bones and joints, on each occasion. The settings on the ultrasound scan equipment and probe were optimised to accurately delineate all the anatomical structures. Although there are no published data on the relationship of cross-sectional area on ultrasound scan and disease progression, the surface area of Dupuytren’s disease has been reported to correlate with extension deficit. Over a period of 20 months, each cm^2^ increase in area was predicted to increase the risk of being in Tubiana grading stage 4 (total passive extension deficit >135°) by an odds ratio of 3· 2.^30^

The data from the most restricted activity indicates that most participants had some difficulty with grip and associated tasks. However, the difficulty with such tasks did not appear to have a significant effect on overall hand function, with the MHQ scoring about 80 in both groups throughout the course of the trial. It is probable that MHQ is not sufficiently sensitive to detect small differences in participants with early-stage Dupuytren’s disease, which has relatively little impairment of hand function, and is subject to a ceiling effect. There was also no change in the most restricted activity selected by each participant. Dupuytren’s disease typically progresses over several years^3,4^ and a limitation of our study is that we only followed patients for 18 months from baseline. Consequently, we did not observe a significant change in the extensor deficit of the joint affected by the treated nodule over the course of the study. Approximately 20% of patients with Dupuytren’s disease progressed to the development of finger contractures over 7 years in one study;^3^ in another study, progression was reported in about 35% patients over the course of 18 years.^4^ Therefore, follow-up for 10 years or more would be required to ascertain whether intranodular injection of adalimumab and the observed significant reduction in nodule hardness and nodule size on ultrasound scan would affect the development of finger deformities and hand function as assessed by patient-reported outcome measures such as MHQ. Predictably, there was no change in grip strength over time in either treatment group. Only a small number of participants, three in the adalimumab and ten in the placebo group, progressed to surgery during the trial or were planning to undergo surgery related to the study nodule at the 18 month timepoint. Follow-up over a much longer period would be required to ascertain whether this trend would be reflected as significant differences although as noted previously, we would suggest that participants might consider another course of four injections of adalimumab if the nodule were to re-activate.

Anti-TNF drugs have been administered to over 10 million people worldwide and adalimumab to approximately 5 million. Adalimumab has an excellent safety record, with the major adverse effects relating to infection or reactivation of latent tuberculosis. We screened all participants for tuberculosis and also applied all the other exclusion criteria used in routine clinical care for patients with systemic inflammatory disorders. There were no related serious adverse events. Minor local injection related reactions occurred on 25 occasions and were unrelated to substance injected.

Another limitation of our study is that we did not collect data on the race and ethnicity of the trial participants (which was not standard practice in 2017).

In conclusion, this phase 2b, randomised trial shows that four injections of 40 mg adalimumab in 0·4 mL at 3 month intervals resulted in reduction in nodule hardness and size, both of which continued to decrease for the duration of the follow-up period, which was 9 months after the last injection. Adalimumab was found to be safe, and the development of anti-adalimumab antibodies did not affect the outcomes. Taken together with our findings that anti-TNF down-regulates the myofibroblast phenotype (α-SMA and collagen type 1 expression and contractility) in vitro,^15,16^ and our earlier phase 2a data showing that 40 mg of adalimumab in 0· 4 mL reduces the expression of α-SMA and procollagen type 1 proteins,^18^ the data from this phase 2b trial suggest that intranodular injections of adalimumab might reduce progression of early-stage Dupuytren’s disease. Follow-up over a period of approximately 10 years would be required to assess the effect on flexion deformity and hand function.

## Supplementary Material

appendix

## Figures and Tables

**Table 1:** Baseline characteristics

	Adalimumab (n=70)	Saline (n=70)
Site		
Oxford	56(80%)	56 (80%)
Edinburgh	14 (20%)	14 (20%)
Female	27 (39%)	20 (29%)
Male	43 (61%)	50 (71%)
Age at randomisation, years	60·2 (9·7)	59·2 (10·3)
Age at onset of Dupuytren’s disease, years	52·9 (12·5)	52·7 (11·9)
Digit affected by treated nodule		
Index	0	2 (3%)
Middle	15 (21%)	6 (9%)
Ring	39 (56%)	39 (56%)
Little	16 (23%)	23 (33%)
Joint affected by treated nodule		
MCP	54 (77%)	60 (86%)
PIP	16 (23%)	10 (14%)
DIP	0	0
Family history, first degree relatives	25 (36%)	30 (43%)
Garrod’s knuckle pads	12 (17%)	19 (27%)
Plantar (Ledderhose) disease	12 (17%)	10 (14%)^*^
Peyronie’s disease	3 (4%)	3 (4%)
Epilepsy	1 (1%)	2 (3%)
Liver disease	0	0
Significant exposure to occupational vibration	4 (6%)	6 (9%)
Type 1 diabetes	0	1 (1%)
Type 2 diabetes	3 (4%)	5 (7%)
Current or previous frozen shoulder	19 (27%)	18 (26%)
Current smoker	4 (6%)	3 (4%)
Previous significant trauma to affected hand	13 (19%)	14 (20%)
Nodule hardness (standard durometer)	63·2 (8·4)	61·4 (9·7)
Nodule area, mm^2^	27·7 (17·6)†	32·2 (22·2)‡
Nodule height, mm	4·2 (1·6)†	4·5 (1·8)‡
Nodule feret, mm	9·8 (3·2)†	10·1 (3·7)‡

Data are n (%) or mean (SD). MCP=metacarpophalangeal. PIP=proximal interphalangeal. DIP=distal interphalangeal.

* Denominator for saline=69.

† Denominator=69.

‡ Denominator=66.

**Table 2:** Primary and key secondary outcome measures

	Adalimumab^*^		Saline^*^		Treatment effect†	
	n	Mean (SD)	n	Mean (SD)	Mean difference	p value
**Standard durometer^‡^**						
Baseline	70	63·2 (8·4)	70	61·4 (9·7)	‥	‥
3 months	67	62·0 (9·2)	65	62·1 (8·9)	-1·6 (-3·8 to 0·7)	0·17
6 months	64	60·7 (10·4)	60	61·2 (10·0)	-2·0 (-4·3 to 0·4)	0·098
9 months	63	58·7 (11·6)	59	62·0 (9·3)	-4·5 (-6·9 to -2·1)	0·0002
12 months	59	58·1 (11·8)	54	61·2 (9·8)	-4·6 (-7·1 to -2·2)	0·0002
18 months	53	55·2 (13·7)	39	60·3 (10·0)	-5·8 (-8·7 to -3·0)	<0·0001
**Nodule area, mm^2^**						
Baseline	69	27·7 (17·6)	66	32·2 (22·2)	‥	‥
12 months	61	21·8 (18·7)	63	35·9 (28·9)	-8·4 (-13·8 to -2·9)	0·0025
18 months	60	18·1 (18·9)	55	34·4 (27·8)	-14·4 (-19·9 to -9·0)	<0·0001
**Nodule height, mm**						
Baseline	69	4·2 (1·6)	66	4·5 (1·8)	‥	‥
12 months	61	3·8 (1·9)	63	4·6 (2·2)	-0·4 (-0·9 to 0·0)	0·064
18 months	60	3·3 (2·1)	55	4·5 (2·2)	-1·1 (-1·5 to -0·6)	<0·0001
**Nodule feret, mm**						
Baseline	69	9·8 (3·2)	66	10·1 (3·7)	‥	‥
12 months	61	7·8 (3·2)	63	10·5 (4·4)	-2·3 (-3·3 to -1·2)	<0·0001
18 months	60	7·0 (3·5)	55	10·3 (5·0)	-3·3 (-4·3 to -2·2)	<0·0001

* Observed data (nodule area, height, and feret) presented without imputation for missing data.

† Treatment effects obtained from multilevel mixed-effects models adjusted for baseline scores, site, and age.

‡ Missing outcome data were handled by multiple imputation by chained equations using predictive mean matching. Participants were analysed by their randomised intervention, regardless of compliance. Missing baseline data were mean imputed in all analysis models. Some durometer readings were missing, especially at later timepoints (10/17 missing values in the adalimumab group, 15/31 missing values in the saline group at 18 months) because disease progression precluded reliable assessment due to the relatively wide base plate of the standard durometer. One nodule hardness outcome in the adalimumab group and seven in the saline group were missing because the relevant nodule had been surgically excised. The surgery for the participant with the nodule treated with adalimumab was for another nodule affecting the same ray. Data missing for one participant in the saline group who received radiotherapy. Data at 18 months (6 in the adalimumab group and 8 in the saline group) were missing for other reasons, including lost to follow-up, withdrawal from trial, unable to attend owing to COVID-19.

**Table 3:** Local adverse events

	Adalimumab	Saline
Local itching	6	4
Redness	3	5
Blister	0	2
Nerve injury	0	0
Local bruising	0	2
Haematoma at injection site	0	3

Data are shown across all timepoints. One adverse event of grade 3 was reported (pericarditis in a participant in the placebo group) over the course of the trial.

## Data Availability

Aggregate data will be shared at the end of the trial with external researchers who provide a methodologically sound proposal to the trial team (and will be required to sign a data sharing access agreement with the sponsor) and in accordance with the guidelines of the sponsor and funders. Study documents including study protocol, statistical analysis plan, and participant consent form can also be made available. Requests for data or study documents should be directed to the corresponding author and will be considered by the chief investigator in conjunction with other members of the trial management group and the trials unit.

## Research in context

### Evidence before this study

We searched Ovid Medline and Embase databases on Nov 1, 2021 using a search strategy which combined relevant thesaurus and free text terms to retrieve articles about interventions for Dupuytren’s disease. No limits were applied. Details of the search strategy are available in our systematic review of non-surgical treatments for early-stage Dupuytren’s disease published in 2016, which suggested intralesional steroid injections or radiotherapy lead to softening of the Dupuytren’s nodules and potential control of disease progression. However, the studies identified in that systematic review were poorly designed, uncontrolled, and neither the participants nor assessors were masked. A randomised, double-blind, placebo-controlled trial of collagenase with 17−22 participants in each treatment group reported reduction in nodule hardness and surface area over an 8-week period. A prospective cohort study found that increase in nodule surface area was correlated with progression of total passive extension deficit over 20 months. Subsequently, in a randomised trial of extracorporeal shockwave therapy in 52 patients, those in the treatment group reported significant reduction in pain scores over 18 months, with no significant changes in other patient reported outcome measures. Our laboratory studies have shown that Dupuytren’s disease is a localised inflammatory disorder. The development and maintenance of myofibroblasts, the cells responsible for deposition and contraction of the collagenous matrix is dependent on the secretion of low amounts of tumour necrosis factor (TNF) by local immune cells, including macrophages and mast cells. In a dose ranging phase 2a clinical trial we found that local injection of anti-TNF (adalimumab 40 mg in 0·4 mL) led to down-regulation of markers of myofibroblast phenotype.

### Added value of this study

Our randomised, controlled trial of four injections at 3 month intervals of adalimumab into the nodules of patients with early-stage Dupuytren’s disease led to softening and reduction in size of the treated nodule, and both these parameters continued to decrease for 9 months after the fourth injection. Patients with early-stage disease have little impairment of hand function and we did not find any change in patient reported outcome measures of hand function (Michigan Hand Questionnaire and most restricted activity). Over the 18-month duration of this trial there was no significant change in passive extension deficit of the affected metacarpophalangeal joints, although the passive extension defecit appeared to be better when nodules that affected proximal interphalangeal joints were treated with adalimumab. However, the number of proximal interphalangeal joints involved was small. Over the course of the trial, more participants in the saline group progressed or were awaiting surgery than in the adalimumab group; again, the overall numbers were small. The treatment was safe and there were no related severe adverse events.

### Implications of all the available evidence

Our in vitro and phase 2a clinical trial data showing that anti-TNF downregulates the myofibroblast phenotype together with the results of this study suggest that intranodular injections of adalimumab might be helpful to control the progression of early-stage Dupuytren’s disease; follow-up over approximately 10 years would be required to confirm this. We would envisage that the patient would undergo a further series of injections if the nodule were to re-activate, and each active nodule would need to be injected.
