# Supplementary material for: Anti-tumour necrosis factor therapy for early-stage Dupuytren’s disease (RIDD): a phase 2b, randomised, double-blind, placebo-controlled trial
Source: Lancet Rheumatol. Author manuscript; Available in PMC 2022 Aug 9. (PMC7613263; doi:10.1016/S2665-9913(22)00093-5)
Supplement: appendix [file EMS151613-supplement-appendix.pdf]

THE LANCET
Rheumatology

Supplementary appendix

This appendix formed part of the original submission and has been peer reviewed. We post it as supplied by the authors.

Supplement to: Nanchahal J, Ball C, Rombach I, et al. Anti-tumour necrosis factor therapy for early-stage Dupuytren's disease (RIDD): a phase 2b, randomised, double-blind, placebo-controlled trial. *Lancet Rheumatol* 2022; published online April 29. https://doi.org/10.1016/S2665-9913(22)00093-5.

SUPPLEMENTAL MATERIAL to "Anti-Tumour Necrosis Factor Therapy for Early Stage Dupuytren's Disease (RIDD): a phase 2b randomised double blind, placebo-controlled trial"

Contents

Supplemental: Changes in analytical methods from those pre-specified in the Protocol and Statistical Analysis Plan 2

Supplemental Table S1: Protocol amendments..... 3

Supplemental: Recruitment from the Netherlands 6

Supplemental Table S2: Comparison of the UK and Netherlands population..... 7

Supplemental Figure S1: Bland Altman plot for durometer measurements 8

Supplemental Table S3: Descriptive summary for the Netherlands trial population 9

Supplemental Table S4: Sensitivity analyses for primary outcome 13

Supplemental Figure S2: Sensitivity analysis for missing data, primary outcome..... 15

Supplemental Figure S3: Forest plot of primary outcome by stratification factor and other prognostic variables 16

Supplemental Figure S4: Representative ultrasound images 17

Supplemental Table S5: Michigan (MHQ) & most restricted activity data 18

Supplemental Table S6: Grip strength and range of movement data 21

Supplemental Figure S5: Pain during and immediately after injection 24

Supplemental Figure S6: Circulating levels of adalimumab..... 25

Supplemental Figure S7: Quantitative assay for antibodies to adalimumab antibodies 26

Supplemental Figure S8: Relationship between the concentration of circulating antibodies to adalimumab (quantitative RIDA screen) and change in durometer readings, nodule area, feret or height 27

Supplemental: Changes in analytical methods from those pre-specified in the Protocol and Statistical Analysis Plan

We analysed the primary endpoint (nodule hardness at 12 months) with a linear mixed effects model including all follow-up time points, adjusted for baseline nodule hardness, age and trial site, instead of the pre-specified ANCOVA model using just the 12 month outcome data and baseline nodule hardness as the only covariate.

We made this change for the following reasons:

1. The mixed model was able to take into account the high correlation between the follow-up data for each participant and, therefore, is a more powerful way of analysing the data.
2. Treatment effects for all follow-up time points could be derived from the mixed model, reducing the number of statistical models required for this analysis.
3. Adjustment for randomisation factors is in line with recommendation for RCT analysis and therefore was preferred as the principal analysis. Analyses without adjustment for the randomisation factors are provided in appendix (p 13) and show consistent results.
4. Results from an ANCOVA model analysing only the 12 month follow-up data were very similar to those from our primary analysis. Treatment effects differ only at the decimal place (-4.5 vs -4.6), and as expected, confidence intervals of the ANCOVA are wider (-7.3, -1.7 vs -7.1, -2.2) because this analysis approach has less power as fewer data are included. Crucially, the interpretation of the clinical and statistical significance of the result remains unchanged.

Supplemental Table S1: Protocol amendments

Amendment number	Protocol Version	Date approved by REC/HRA	Changes
1	3.4	30 Jul 2015/NA	Part 1: criteria for progression to next dose; unblinding; randomisation procedure; removal of DASH and hand therapy record; addition of Ametop as nIMP; investigator added; visit time windows broadened; screening procedures for anti-TNF amended to be consistent with those used in standard clinical practice; addition of surgery site assessment.
2	4.0	14 Sep 2015/NA	Part 1 and 2: Amend time for avoidance of pregnancy in line with SmPC; amend unblinding to office hours; GP letter with PI details
3	5.0	08 Jan 2016/NA	Part 1: reduction of secondary outcomes and visits to reduce patient burden.<br>Part 2: add blood sample at 12 months
4	6.0	15Mar 2016/NA	Part 1: Remove safety and run-in from title; permit dose cohorts in different order; randomise using RRAMP
5	7.0	11 May 2016/19 Jun 2016	Part 1: remove systemic administration adalimumab<br>Part 2: telephone call 1 wk after injection instead of visit, clarification of recruitment of patients with clear history of progression of Dupuytren's disease; exclude patients on coumarin anti-coagulants and systemic steroids
6	n/a	13 Jun 2016/15 Jul 2016	Include additional recruitment site (Queen Victoria Hospital, East Grinstead)
7	8.0	25 Oct 2016/ 21 Nov 2016	Part 1: addition of 40mg adalimumab in 0.4ml dose cohort; addition of α-SMA protein levels as outcome measure

			Part 1 & 2: remove assessment of vascularity using colour Doppler ultrasound; remove assessment of heart rate and BP at clinic attendance.
8	n/a	21 Feb 2017	Change web address and telephone number on poster
Email exchange/ TMG Meeting	n/a	April 2015 – Jun 2017<br><br><br><br><br><br>Oct 2017 – Apr 2018	Queen Victoria hospital, East Grinstead, declined to participate due to lack of research infrastructure/support<br><br>Decision by research team to approach Groningen, NL as high local prevalence of Dupuytren's disease and existing cohort of patients with early-stage disease being monitored for disease progression<br>Recruitment in 2 UK centres (Oxford and Edinburgh) slower than predicted
9	n/a	NA/05 Jul 2017	Part 2: Update resource use questionnaire
10	n/a	NA/11 Jul 2017	Part 2: Update GP poster
11	n/a	NA /07Sep2017	Part 2: Update resource use questionnaire
12	9.0	11Jan2018/ 24Jan2018	Part 1 and 2: addition of exploratory objectives to blood and tissue samples.<br>Part 2: define primary objective as nodule hardness at 12 months, other time points as secondary objectives; addition of injection experience (pain) as secondary objective; add resource use as tertiary objective; exclude thumbs and previous radiotherapy to hand and surgery/collagenase/steroid to ray; increase geographical areas for recruitment in UK via posters in GP surgeries; increase interval between screening and baseline visit to 12 wk; increase windows for follow up to ± 4wk
13	n/a	NA/02Mar 2018	Part 2: Reformat poster

14	10.0	11 Jul 2018/18 Jul 2018	Part 1: Remove parenteral steroid as exclusion criteria
15	11.0	16Oct2018/16Nov2018	Part 2: Change to allow minimum target recruitment of 138 to maximum of 200.<br>Remove scheduled elective surgery or other procedures requiring general anaesthesia during the study as exclusion criterion
16	n/a	10Sep2018	Part 2: GDPR Implementation
17	12.0	03Sep2019/04Sep2019	Part 2: enable new markers or assays relevant to Dupuytren's disease as they become known/available for the tissue and/or blood samples collected as part of the trial
18	13.0	NA/06Dec2019	Part 2: include all interventions that may take place during the trial instead of limiting to surgical excision of the treated nodule.

Supplemental: Recruitment from the Netherlands

We screened 48 and recruited 33 participants from the University Medical Centre Groningen (UMCG), Netherlands between February 2019 to April 2019. We anticipated that the patients in the Netherlands may have a more aggressive disease phenotype (appendix p 7), and since we experienced difficulty using the standard probe durometer in UK participants when disease progressed, a slim probe durometer (Rex Gauge RX-1600-OO) was used in the Netherlands. From August 2018 we measured nodule hardness using both standard and slim probe durometers in the UK participants. Consequently, baseline data using the slim durometer were only available for a limited number of UK participants. Our plan was to combine the data from the standard and slim durometers using cross-walk methodology[1] to map the results of the standard to the slim durometer. Blinded analysis of the durometer data showed high prediction error and insufficient overlap between the samples[2] and hence a reliable cross-walk could not be performed (appendix p 8). Furthermore, durometer readings could not be obtained for the majority of the Netherlands participants at 18 months due to the COVID-19 pandemic. Therefore, descriptive analyses of the subsample recruited in the Netherlands are presented (appendix p 9-12). There was a similar trend with nodule hardness being reduced in the adalimumab group but no statistical analyses are presented due to the low numbers. Ultrasound examination in the Netherlands was performed using a Esaote MyLabOne device with SL3235 probe (depth 2 cm, 18 MHz). However, the low quality of the ultrasound scan images precluded assessment by the blinded observer (CB) who assessed all the scans from the UK participants.

Supplemental Table S2: Comparison of the UK and Netherlands population

	UK (n=140)	NL (n=33)
**Female**	47 (34%)	16 (48%)
**Age at randomisation (yr)**	59.7 (10.0)	56.9 (9.5)
**Age at onset of Dupuytren's Disease (yr)**	52.8 (12.2)	45.3 (14.5)
**Current smoker**	7 (5%)	5 (15%)
**Epilepsy**	3 (2%)	0 (0%)
**Liver disease**	0 (0%)	1 (3%)
**Significant exposure to occupational vibration**	10 (7%)	2 (6%)
**Previous significant trauma to affected hand**	27 (19%)	6 (18%)
**Type 1 diabetes**	1 (1%)	1 (3%)
**Type 2 diabetes**	8 (6%)	2 (6%)
**Frozen shoulder***	37 (26%)	13 (39%)
**Plantar (Ledderhose's) disease***	22 (16%)$^{+}$	15 (45%)
**Peyronie's disease***	6 (4%)	2 (6%)
**Garrod's knuckle pads***	31 (22%)	7 (21%)
**Family history (1st degree relatives)***	65 (46%)	25 (76%)

Characteristics associated with Dupuytren's diathesis[3]. Current or previous frozen shoulder. Standard deviations for age

$^{+}$*Denominator 139*

Supplemental Figure S1: Bland Altman plot for durometer measurements

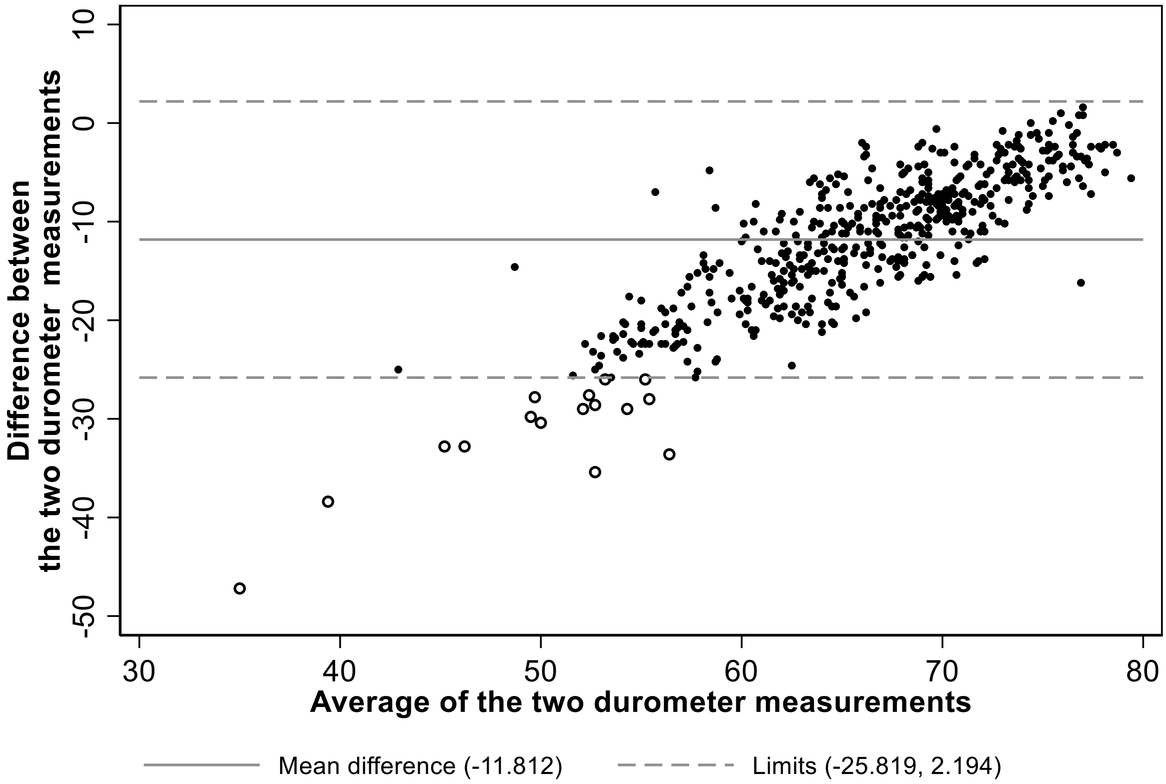

Note: Outliers are 16 different participants across all follow-up timepoints

Bland-Altman plot[4] showing the mean of the two durometer measurements against the difference between the two measurements. The plot shows very limited agreement between the two durometer measurements, and that the level of disagreement is larger for lower durometer readings. Detailed interpretation: The mean of the differences (shown on the y-axis) between the two durometer reading was -11.812 (grey solid horizontal line), indicating that on average, the standard durometer produces a measurement that is almost 12 point lower than the measurement generated by the slim probe durometer. For measurements with good agreement, this mean difference would be expected to be close to zero, indicating very similar results are obtained using the different measures. Also, differences between the two durometer measurements would be distributed randomly across the average of the two durometer measurements (x-axis). This means that we would not expect to see a pattern of points between the two dashed lines, indicating that the size of the measurement error does not depend on the durometer reading itself. In the plot above, the individual observations form a diagonal line which indicates that the two durometers are more likely to produce similar results for higher durometer readings but are more likely to generate very different readings for lower durometer readings. In conclusion, the Bland-Altman plot indicates that there is inconsistency in the results the two durometers provide, and that discrepancies are higher for lower durometer readings.

Supplemental Table S3: Descriptive summary for the Netherlands trial population

	Adalimumab[1]		Saline[1]	
	n	Mean (SD)	n	Mean (SD)
**Slim durometer**				
**Baseline**	16	68.8 (3.9)	17	71.7 (2.6)
**3 months**	16	67.0 (6.3)	17	70.9 (2.8)
**6 months**	16	66.9 (6.3)	16	70.4 (5.1)
**9 months**	15	67.7 (4.7)	14	70.2 (3.3)
**12 months**	14	68.6 (4.3)	13	71.5 (3.7)
**18 months**	3	64.5 (6.0)	3	71.4 (7.5)
**MHQ for treated hand**				
**Baseline**	16	82.8 (8.3)	17	70.9 (21.3)
**3 months**	16	87.7 (10.9)	17	75.0 (20.9)
**6 months**	16	90.0 (8.6)	17	74.4 (19.1)
**9 months**	16	87.7 (8.9)	16	75.0 (21.2)
**12 months**	16	87.4 (9.7)	17	73.6 (19.3)
**18 months**	16	86.2 (10.1)	17	69.6 (17.8)
**MHQ - overall hand function**				
**Baseline**	16	75.9 (9.7)	17	66.5 (24.7)
**3 months**	16	78.1 (13.0)	17	68.9 (21.4)
**6 months**	16	81.6 (12.1)	17	66.5 (21.0)
**9 months**	16	84.3 (12.3)	16	68.8 (24.0)
**12 months**	16	77.2 (14.1)	17	69.7 (23.0)
**18 months**	16	74.7 (15.0)	17	62.6 (20.8)
**MHQ - activities of daily living**				
**Baseline**	16	95.1 (6.2)	17	83.2 (18.8)
**3 months**	16	97.2 (4.7)	17	85.7 (19.0)
**6 months**	16	96.2 (4.7)	17	86.9 (16.0)
**9 months**	16	97.3 (4.1)	16	83.7 (17.1)
**12 months**	16	97.3 (4.3)	17	84.4 (16.9)
**18 months**	16	96.5 (4.6)	17	80.4 (17.2)
**MHQ - work performance**				
**Baseline**	16	89.1 (12.3)	17	77.6 (28.1)
**3 months**	16	94.4 (12.2)	17	78.8 (27.5)
**6 months**	16	95.3 (9.2)	17	84.4 (18.4)

	Adalimumab[1]		Saline[1]	
	n	Mean (SD)	n	Mean (SD)
**9 months**	16	96.6 (7.9)	16	81.9 (25.0)
**12 months**	16	94.7 (9.0)	17	80.6 (24.9)
**18 months**	16	95.3 (6.9)	17	77.4 (26.3)
**MHQ - pain**				
**Baseline**	16	73.8 (12.0)	17	61.9 (19.7)
**3 months**	16	87.9 (14.7)	17	72.8 (21.6)
**6 months**	16	90.0 (13.9)	17	70.0 (21.7)
**9 months**	16	83.7 (12.8)	15	76.1 (23.9)
**12 months**	16	87.1 (12.7)	17	70.3 (22.3)
**18 months**	16	91.1 (11.9)	17	67.9 (20.3)
**MHQ - aesthetics**				
**Baseline**	16	86.7 (14.2)	17	73.9 (21.0)
**3 months**	16	86.7 (19.7)	17	78.7 (19.3)
**6 months**	16	89.5 (13.8)	17	72.4 (23.2)
**9 months**	16	86.7 (15.3)	16	68.8 (23.4)
**12 months**	16	87.5 (13.9)	17	72.1 (20.9)
**18 months**	16	87.5 (17.4)	17	72.1 (19.0)
**MHQ - patient satisfaction with hand function**				
**Baseline**	16	76.3 (17.9)	17	62.0 (30.7)
**3 months**	16	82.0 (17.9)	17	65.2 (31.7)
**6 months**	16	87.5 (16.5)	17	65.9 (29.2)
**9 months**	16	77.6 (25.0)	16	70.8 (30.1)
**12 months**	16	81.0 (20.1)	17	64.5 (33.2)
**18 months**	16	72.1 (29.6)	17	57.5 (29.9)
**Most restricted activity rating**				
**Baseline**	15	6.7 (1.5)	16	5.4 (2.3)
**3 months**	11	6.1 (1.3)	17	6.1 (2.5)
**6 months**	15	7.3 (1.8)	17	5.8 (2.3)
**9 months**	14	7.6 (1.7)	16	6.4 (2.8)
**12 months**	11	7.5 (2.3)	17	6.1 (2.2)
**18 months**	6	8.2 (1.9)	13	7.1 (2.1)

	Adalimumab[1]		Saline[1]	
	n	Mean (SD)	n	Mean (SD)
**Mean of grip strength measurements (kg)**				
**Baseline**	16	38.1 (11.5)	17	32.2 (12.0)
**3 months**	16	38.6 (11.9)	17	31.9 (10.6)
**6 months**	16	39.6 (9.2)	16	32.2 (11.2)
**9 months**	15	39.2 (10.6)	14	32.5 (12.6)
**12 months**	14	38.5 (10.9)	13	33.4 (11.1)
**18 months**	3	43.8 (12.9)	3	28.0 (4.2)
**Overall active extension deficit of joint affected by treated nodule (degrees)**				
**Baseline**	16	5.4 (13.0)	17	6.8 (15.1)
**3 months**	16	4.7 (16.5)	17	12.1 (18.7)
**6 months**	16	6.1 (19.4)	16	9.4 (19.0)
**9 months**	15	6.0 (21.7)	14	10.4 (16.5)
**12 months**	14	8.0 (14.8)	13	13.5 (20.8)
**18 months**	3	13.3 (25.2)	3	20.7 (16.2)
**MCP: active extension deficit of joint affected by treated nodule (degrees)**				
**Baseline**	13	4.3 (13.6)	16	5.4 (14.5)
**3 months**	13	2.6 (17.1)	16	11.1 (18.9)
**6 months**	13	2.5 (18.1)	15	7.3 (17.7)
**9 months**	13	3.8 (21.1)	14	10.4 (16.5)
**12 months**	12	6.0 (12.5)	13	13.5 (20.8)
**18 months**	2	0.0 (14.1)	3	20.7 (16.2)
**PIP: active extension deficit of joint affected by treated nodule (degrees)**				
**Baseline**	3	10.3 (11.1)	1	28.0 (n/a)
**3 months**	3	13.7 (11.8)	1	28.0 (n/a)
**6 months**	3	21.7 (20.2)	1	41.0 (n/a)
**9 months**	2	20.0 (28.3)	0	n/a

	Adalimumab[1]		Saline[1]	
	n	Mean (SD)	n	Mean (SD)
**12 months**	2	20.0 (28.3)	0	n/a
**18 months**	1	40.0 (n/a)	0	n/a
**Overall passive extension deficit of joint affected by treated nodule (degrees)**				
**Baseline**	16	2.3 (5.5)	17	3.4 (7.1)
**3 months**	16	0.6 (2.5)	17	5.4 (11.2)
**6 months**	16	2.5 (5.8)	16	3.9 (9.0)
**9 months**	15	0.7 (1.8)	14	3.9 (7.4)
**12 months**	14	1.4 (5.3)	13	5.4 (7.8)
**18 months**	3	10.0 (17.3)	3	13.3 (11.5)
**MCP: passive extension deficit of joint affected by treated nodule (degrees)**				
**Baseline**	13	1.5 (5.5)	16	2.2 (5.5)
**3 months**	13	0.0 (0.0)	16	4.5 (10.9)
**6 months**	13	1.5 (3.8)	15	2.1 (5.8)
**9 months**	13	0.4 (1.4)	14	3.9 (7.4)
**12 months**	12	0.0 (0.0)	13	5.4 (7.8)
**18 months**	2	0.0 (0.0)	3	13.3 (11.5)
**PIP: passive extension deficit of joint affected by treated nodule (degrees)**				
**Baseline**	3	5.3 (5.0)	1	22.0 (n/a)
**3 months**	3	3.3 (5.8)	1	20.0 (n/a)
**6 months**	3	6.7 (11.5)	1	30.0 (n/a)
**9 months**	2	2.5 (3.5)	0	n/a
**12 months**	2	10.0 (14.1)	0	n/a
**18 months**	1	30.0 (n/a)	0	n/a

Supplemental Table S4: Sensitivity analyses for primary outcome

	Adalimumab[1]		Saline[1]		Treatment effect - available cases				Treatment effect - imputed data			
					unadjusted		adjusted		unadjusted		adjusted	
	n	Mean (SD)	n	Mean (SD)	Difference*	p-value	Difference*	p-value	Difference*	p-value	Difference*	p-value
**Standard durometer – ITT**												
**Baseline**	70	63.2 (8.4)	70	61.4 (9.7)								
**3 months**	67	62.0 (9.2)	65	62.1 (8.9)	-1.7 (-3.9, 0.4)	0.11	-1.7 (-3.8, 0.5)	0.12	-1.6 (-3.9, 0.7)	0.16	-1.6 (-3.8, 0.7)	0.17
**6 months**	64	60.7 (10.4)	60	61.2 (10.0)	-2.1 (-4.3, 0.0)	0.055	-2.1 (-4.3, 0.1)	0.062	-2.0 (-4.4, 0.3)	0.091	-2.0 (-4.3, 0.4)	0.098
**9 months**	63	58.7 (11.6)	59	62.0 (9.3)	-4.4 (-6.6, -2.2)	<0.0001	-4.3 (-6.5, -2.1)	0.0001	-4.5 (-6.9, -2.2)	0.00018	-4.5 (-6.9, -2.1)	0.0002
**12 months**	59	58.1 (11.8)	54	61.2 (9.8)	-3.7 (-6.0, -1.5)	0.001	-3.7 (-5.9, -1.5)	0.0012	-4.6 (-7.1, -2.2)	0.00021	-4.6 (-7.1, -2.2)	0.00024
**18 months**	53	55.2 (13.7)	39	60.3 (10.0)	-6.2 (-8.6, -3.9)	<0.0001	-6.2 (-8.6, -3.8)	<0.0001	-5.9 (-8.8, -3.0)	<0.0001	-5.8 (-8.7, -3.0)	<0.0001
**Standard durometer - PP**												
**Baseline**	60	63.1 (8.7)	58	61.0 (10.0)								
**3 months**	58	61.9 (9.1)	57	61.7 (9.0)	-1.7 (-4.0, 0.5)	0.13	-1.7 (-3.9, 0.5)	0.14	-1.6 (-3.9, 0.8)	0.19	-1.5 (-3.8, 0.8)	0.21
**6 months**	59	60.5 (10.3)	52	60.5 (10.2)	-1.9 (-4.2, 0.3)	0.092	-1.9 (-4.1, 0.4)	0.10	-2.0 (-4.3, 0.4)	0.10	-1.9 (-4.2, 0.4)	0.11
**9 months**	59	58.7 (11.1)	53	61.6 (9.5)	-4.2 (-6.4, -1.9)	0.00028	-4.1 (-6.4, -1.9)	0.00029	-4.3 (-6.7, -2.0)	0.0003	-4.3 (-6.6, -1.9)	0.00033
**12 months**	56	58.4 (10.4)	48	60.5 (10.0)	-3.5 (-5.8, -1.2)	0.0033	-3.4 (-5.7, -1.1)	0.0035	-4.3 (-6.7, -1.8)	0.00059	-4.2 (-6.6, -1.8)	0.00065
**18 months**	50	55.4 (12.3)	37	60.4 (10.3)	-6.0 (-8.4, -3.6)	<0.0001	-6.0 (-8.4, -3.6)	<0.0001	-5.9 (-8.6, -3.3)	<0.0001	-5.9 (-8.5, -3.2)	<0.0001
**Standard durometer – CACE analysis**												
**12 months**					-3.3 (-4.3, -2.3)	<0.0001	-4.2 (-7.1, -1.3)	0.0044				

**Standard durometer – COVID-19 adjustment**[2]												
**Baseline**	70	63.2 (8.4)	70	61.4 (9.7)								
**3 months**	67	62.0 (9.2)	65	62.1 (8.9)	-1.7 (-3.8, 0.3)	0.099	-1.7 (-3.8, 0.4)	0.11	-1.6 (-3.8, 0.6)	0.14	-1.6 (-3.8, 0.6)	0.15
**6 months**	64	60.7 (10.4)	60	61.2 (10.0)	-2.2 (-4.3, 0.0)	0.046	-2.1 (-4.2, 0.0)	0.051	-2.0 (-4.3, 0.2)	0.079	-2.0 (-4.3, 0.3)	0.084
**9 months**	63	58.7 (11.6)	59	62.0 (9.3)	-4.4 (-6.5, -2.3)	<0.0001	-4.4 (-6.5, -2.2)	<0.0001	-4.6 (-6.9, -2.3)	0.00011	-4.5 (-6.8, -2.2)	0.00012
**12 months**	55	58.1 (12.1)	51	61.0 (10.0)	-3.7 (-5.9, -1.5)	0.00099	-3.7 (-5.9, -1.5)	0.0011	-4.6 (-7.1, -2.2)	0.0002	-4.6 (-7.0, -2.2)	0.00022
**18 months**	33	54.3 (14.6)	26	61.3 (9.8)	-6.4 (-9.1, -3.8)	<0.0001	-6.4 (-9.0, -3.8)	<0.0001	-6.1 (-9.4, -2.8)	0.00037	-6.0 (-9.3, -2.7)	0.00041
**Standard durometer – Surgery adjustment**[3]												
**Baseline**	67	63.3 (8.3)	63	61.0 (9.9)								
**3 months**	64	62.0 (8.8)	59	61.6 (8.9)	-1.6 (-3.7, 0.6)	0.15	-1.5 (-3.6, 0.6)	0.17	-1.5 (-3.7, 0.8)	0.21	-1.4 (-3.7, 0.9)	0.23
**6 months**	61	60.8 (10.3)	54	60.5 (10.1)	-1.7 (-3.9, 0.5)	0.12	-1.7 (-3.8, 0.5)	0.14	-1.6 (-4.0, 0.7)	0.17	-1.6 (-3.9, 0.8)	0.18
**9 months**	61	59.1 (11.1)	55	61.5 (9.4)	-3.8 (-6.0, -1.7)	0.00057	-3.8 (-6.0, -1.6)	0.00067	-4.0 (-6.3, -1.6)	0.00092	-3.9 (-6.3, -1.6)	0.001
**12 months**	57	58.5 (10.3)	50	60.5 (9.8)	-3.2 (-5.4, -1.0)	0.005	-3.1 (-5.4, -0.9)	0.0057	-4.0 (-6.4, -1.6)	0.0012	-3.9 (-6.3, -1.5)	0.0013
**18 months**	52	56.0 (12.4)	39	60.3 (10.0)	-5.5 (-7.8, -3.2)	<0.0001	-5.4 (-7.8, -3.1)	<0.0001	-5.3 (-7.9, -2.6)	<0.0001	-5.2 (-7.8, -2.6)	0.00011

**Differences shown for adalimumab vs. saline*

The per-protocol analysis used the imputation model generated for the primary analysis (i.e. no separate imputation models were run using only participants included in the per-protocol population).

Note: unadjusted differences include baseline values as covariates; adjusted differences include baseline values, site and age as covariates

[1]*Observed data presented without imputation for missing data*

[2]*COVID-19 adjustment: Excludes participants whose 12 and 18 month assessments were more than one month delayed.*

[3]*Surgery adjustment: Excludes participants who had surgery during the trial follow-up*

Acronyms: CACE – complier average causal effects analysis; ITT – intention to treat population; PP – per protocol population

Supplemental Figure S2: Sensitivity analysis for missing data, primary outcome

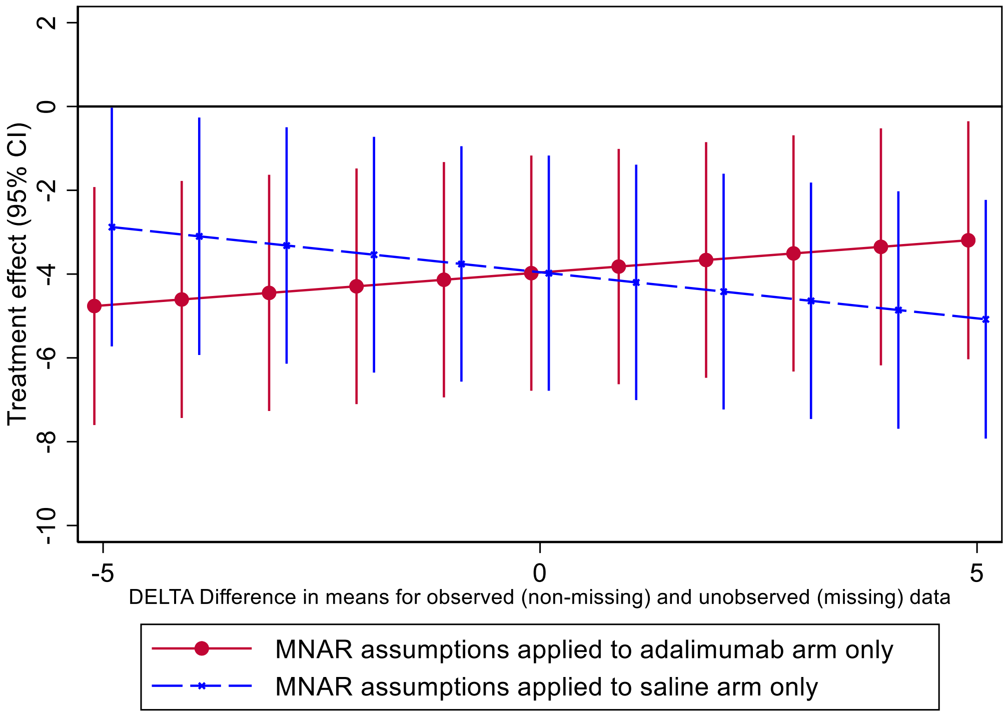

Figure 1: Missing not at random sensitivity analysis (standard durometer) data

This sensitivity analysis investigates the effect on the treatment effects if participants with missing outcome data had nodule hardness measurements (standard durometer) that were, on average, up to 5 units higher or lower than those with observed data.

This model uses only the 12-month follow-up data, and a linear regression model adjusted for baseline durometer values, age, and site.

The data at the x-axis value of 0 show the treatment effects when the available data are assumed to be representative of participants with missing outcomes (i.e. on average, no difference in outcomes is assumed between those with available and those with missing data). The data at the x-axis value of 5 show how the treatment effect would change if it is assumed that the outcomes for adalimumab participants with missing outcome data were 5 points higher (i.e. more nodule hardness and worse outcomes), on average, than those with observed data in the randomised arm (red line). The y-axis shows the treatment effects (i.e. differences in standard durometer results between the treatment arms) for each of the different scenarios considered.

The treatment effects and corresponding CIs remain below 0 in all scenarios, indicating that the conclusions drawn from the trial do not change for any of the scenarios investigated. Therefore, our results are robust to missing data assumptions made in the main analyses.

Abbreviation: CI – confidence interval; MNAR – missing not at random.

Supplemental Figure S3: Forest plot of primary outcome by stratification factor and other prognostic variables

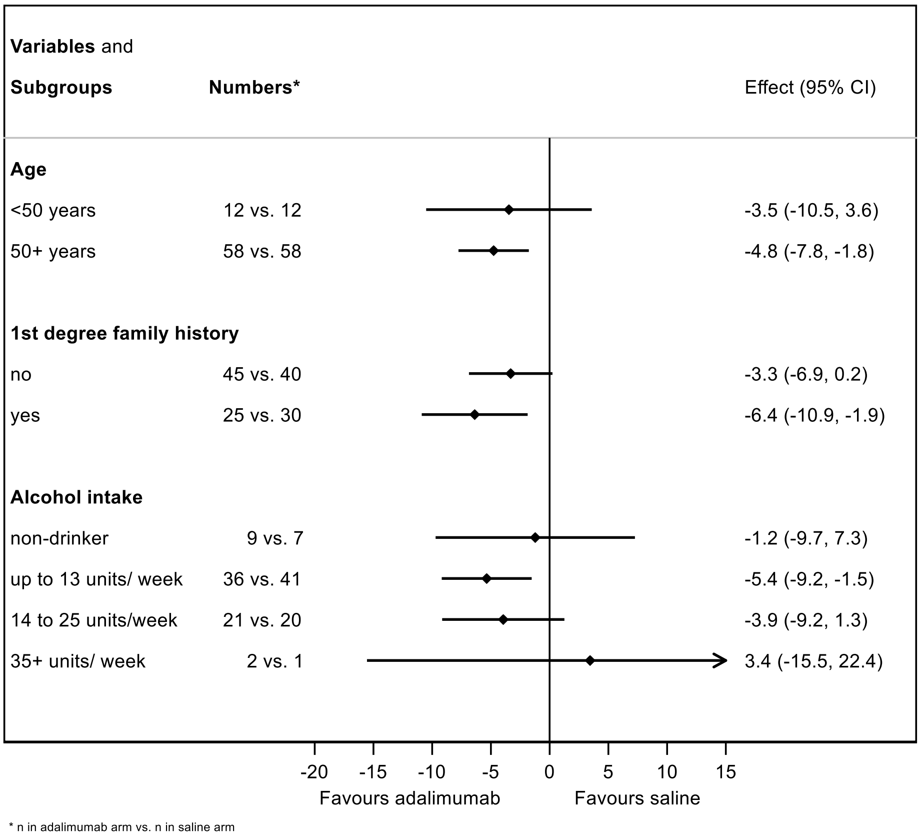

Treatment effects for the subgroups were derived from ANCOVA models adjusted for interactions between the relevant subgroup and the treatment variable. Multiple imputations were used in line with the primary analysis.

Supplemental Figure S4: Representative ultrasound images

Saline

Baseline | 12 months | 18 months

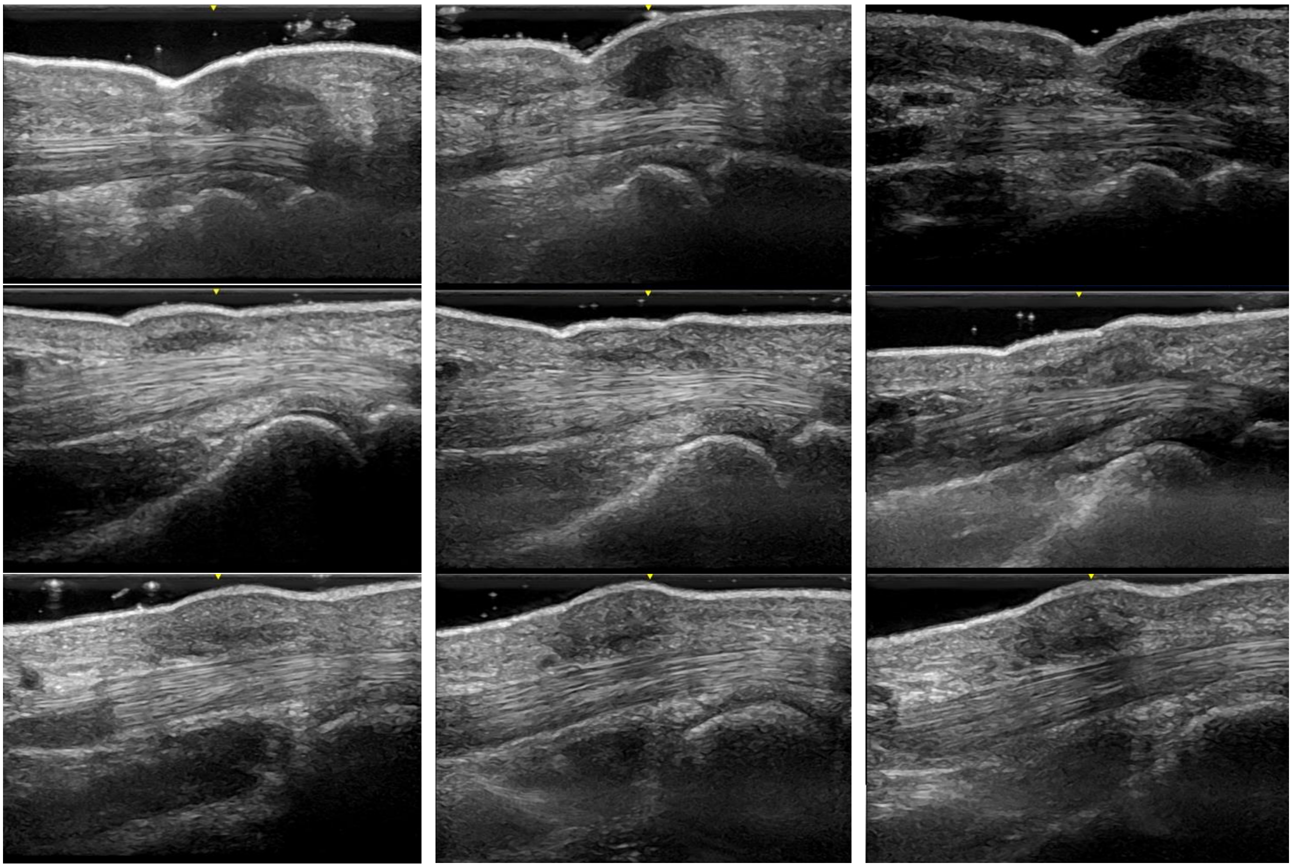

Adalimumab

Baseline | 12 months | 18 months

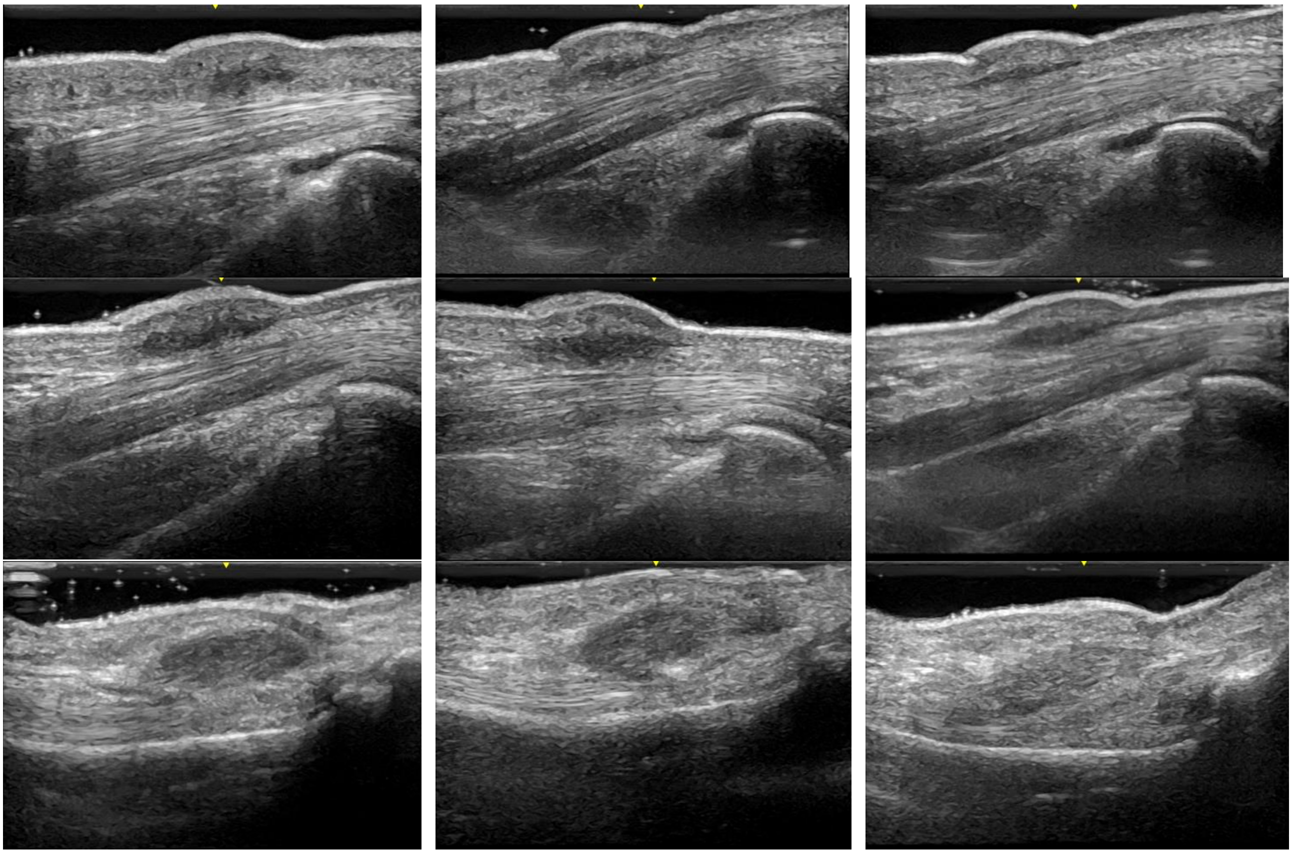

Supplemental Table S5: Michigan (MHQ) & most restricted activity data

	Adalimumab (n=70)		Saline (n=70)		Unadjusted treatment effect*		Adjusted treatment effect*	
	n	Mean (SD)[1]	n	Mean (SD)[1]	Difference (95% CI)	p-value	Difference (95% CI)	p-value
**MHQ for treated hand**								
**Baseline**	70	84.9 (11.7)	70	81.4 (12.3)				
**3 months**	68	86.0 (12.5)	66	81.8 (12.2)	1.6 (-1.8, 5.0)	0.36	1.7 (-1.8, 5.1)	0.34
**6 months**	65	85.8 (13.0)	65	83.0 (13.6)	0.4 (-3.0, 3.9)	0.80	0.5 (-3.0, 3.9)	0.79
**9 months**	64	86.2 (10.9)	66	82.1 (13.6)	1.2 (-2.2, 4.7)	0.48	1.3 (-2.2, 4.7)	0.47
**12 months**	64	84.7 (12.2)	66	81.2 (13.9)	0.6 (-2.8, 4.1)	0.72	0.7 (-2.8, 4.1)	0.71
**18 months**	65	84.4 (11.7)	64	83.1 (14.8)	-1.5 (-5.0, 2.0)	0.40	-1.5 (-4.9, 2.0)	0.41
**MHQ - overall hand function**								
**Baseline**	70	83.6 (14.9)	70	81.9 (16.8)				
**3 months**	68	84.7 (15.2)	66	80.4 (15.2)	3.3 (-0.9, 7.5)	0.13	3.6 (-0.6, 7.7)	0.095
**6 months**	64	83.9 (16.3)	65	81.6 (16.1)	1.3 (-3.0, 5.5)	0.56	1.5 (-2.8, 5.7)	0.49
**9 months**	64	83.9 (15.1)	66	78.3 (16.4)	4.1 (-0.2, 8.4)	0.059	4.3 (0.1, 8.5)	0.045
**12 months**	64	83.5 (16.1)	66	78.8 (16.0)	3.1 (-1.2, 7.3)	0.16	3.3 (-1.0, 7.5)	0.13
**18 months**	65	82.6 (15.2)	64	79.7 (15.7)	1.4 (-2.9, 5.7)	0.52	1.6 (-2.6, 5.8)	0.46
**MHQ - activities of daily living**								
**Baseline**	70	93.8 (7.5)	70	94.0 (7.4)				
**3 months**	68	93.5 (10.4)	66	94.0 (8.7)	-0.6 (-3.8, 2.5)	0.69	-0.6 (-3.7, 2.6)	0.73
**6 months**	65	92.6 (11.4)	65	92.9 (9.3)	-0.2 (-3.4, 3.0)	0.91	-0.1 (-3.3, 3.1)	0.95
**9 months**	63	92.5 (11.2)	66	93.0 (9.5)	-0.2 (-3.4, 2.9)	0.88	-0.2 (-3.3, 3.0)	0.92
**12 months**	64	92.3 (13.5)	66	91.9 (11.9)	0.4 (-2.8, 3.5)	0.83	0.4 (-2.7, 3.6)	0.79
**18 months**	65	92.6 (11.4)	64	91.9 (11.8)	0.6 (-2.6, 3.7)	0.73	0.6 (-2.6, 3.8)	0.70

	Adalimumab (n=70)		Saline (n=70)		Unadjusted treatment effect*		Adjusted treatment effect*	
	n	Mean (SD)[1]	n	Mean (SD)[1]	Difference (95% CI)	p-value	Difference (95% CI)	p-value
**MHQ - work performance**								
**Baseline**	70	92.4 (11.7)	70	88.2 (17.8)				
**3 months**	68	91.5 (16.3)	66	88.9 (14.6)	0.4 (-4.3, 5.1)	0.87	0.5 (-4.3, 5.2)	0.85
**6 months**	65	93.8 (11.9)	65	89.3 (15.8)	2.3 (-2.4, 7.1)	0.34	2.3 (-2.4, 7.1)	0.34
**9 months**	63	94.0 (11.4)	66	91.9 (13.7)	-0.5 (-5.2, 4.3)	0.85	-0.4 (-5.2, 4.3)	0.86
**12 months**	64	92.2 (12.9)	66	87.2 (21.3)	2.5 (-2.3, 7.3)	0.30	2.5 (-2.3, 7.3)	0.30
**18 months**	65	92.4 (14.0)	64	90.4 (15.3)	-0.7 (-5.5, 4.0)	0.76	-0.7 (-5.5, 4.1)	0.77
**MHQ - pain**								
**Baseline**	69	80.4 (18.0)	70	76.2 (18.6)				
**3 months**	68	82.7 (17.6)	65	78.4 (17.9)	2.3 (-2.7, 7.4)	0.37	2.5 (-2.5, 7.4)	0.33
**6 months**	65	83.8 (18.7)	65	81.5 (19.1)	0.9 (-4.2, 6.0)	0.72	0.9 (-4.1, 6.0)	0.72
**9 months**	64	82.6 (16.0)	66	77.5 (18.0)	3.2 (-1.9, 8.3)	0.22	3.2 (-1.9, 8.2)	0.22
**12 months**	63	80.6 (16.9)	66	78.2 (17.6)	0.8 (-4.3, 5.9)	0.75	0.8 (-4.2, 5.9)	0.75
**18 months**	65	82.3 (16.8)	64	80.1 (18.1)	0.6 (-4.5, 5.7)	0.81	0.6 (-4.5, 5.6)	0.82
**MHQ - aesthetics**								
**Baseline**	69	78.7 (18.9)	70	75.5 (20.5)				
**3 months**	68	80.6 (19.1)	66	73.5 (22.0)	4.4 (-1.4, 10.3)	0.14	4.6 (-1.3, 10.4)	0.13
**6 months**	65	78.4 (21.1)	65	76.0 (20.8)	0.5 (-5.5, 6.4)	0.88	0.5 (-5.4, 6.5)	0.86
**9 months**	62	80.8 (19.1)	66	78.7 (20.6)	0.0 (-6.0, 5.9)	0.99	0.0 (-5.9, 6.0)	0.99
**12 months**	64	78.6 (21.0)	66	77.4 (21.5)	-1.5 (-7.4, 4.4)	0.62	-1.4 (-7.4, 4.5)	0.63
**18 months**	65	78.8 (19.7)	64	79.7 (22.5)	-3.0 (-9.0, 2.9)	0.32	-3.0 (-8.9, 3.0)	0.33

	Adalimumab (n=70)		Saline (n=70)		Unadjusted treatment effect*		Adjusted treatment effect*	
	**n**	**Mean (SD)[1]**	**n**	**Mean (SD)[1]**	**Difference (95% CI)**	**p-value**	**Difference (95% CI)**	**p-value**
**MHQ - patient satisfaction with hand function**								
**Baseline**	70	79.9 (21.6)	70	72.4 (24.3)				
**3 months**	67	83.0 (20.0)	65	76.2 (24.1)	3.7 (-3.0, 10.5)	0.28	3.6 (-3.2, 10.4)	0.30
**6 months**	65	82.0 (19.8)	65	76.8 (22.1)	2.0 (-4.8, 8.8)	0.56	1.9 (-4.9, 8.7)	0.59
**9 months**	64	82.7 (19.2)	66	73.4 (25.8)	5.7 (-1.1, 12.4)	0.10	5.5 (-1.3, 12.3)	0.11
**12 months**	64	80.9 (19.2)	65	73.7 (23.6)	2.9 (-3.9, 9.7)	0.40	2.8 (-4.0, 9.6)	0.42
**18 months**	65	77.5 (22.3)	64	77.0 (23.8)	-3.2 (-10.0, 3.6)	0.35	-3.3 (-10.2, 3.5)	0.34
**Most restricted activity rating**								
**Baseline**	67	7.2 (2.3)	69	7.0 (2.1)				
**3 months**	59	7.6 (2.3)	64	6.8 (2.2)	0.5 (-0.2, 1.2)	0.16	0.5 (-0.2, 1.2)	0.14
**6 months**	57	7.4 (2.5)	63	7.0 (2.2)	0.1 (-0.6, 0.8)	0.80	0.1 (-0.6, 0.8)	0.79
**9 months**	61	7.4 (2.5)	63	7.3 (2.3)	0.0 (-0.7, 0.7)	0.93	0.0 (-0.7, 0.7)	0.91
**12 months**	61	7.4 (2.6)	63	6.8 (2.4)	0.4 (-0.3, 1.1)	0.31	0.4 (-0.3, 1.1)	0.30
**18 months**	64	7.3 (2.4)	61	7.2 (2.0)	-0.1 (-0.8, 0.6)	0.88	0.0 (-0.7, 0.7)	0.91

**adjusted differences: adjusted for baseline scores, site, age*
**unadjusted differences: only adjusted for baseline scores*
Missing baseline data were mean imputed in statistical model
[1]Observed data are presented without imputation for missing data

Supplemental Table S6: Grip strength and range of movement data

	Adalimumab (n=70)		Saline (n=70)		Unadjusted treatment effect*		Adjusted treatment effect*	
	n	Mean (SD)[1]	n	Mean (SD)[1]	Difference (95% CI)	p-value	Difference (95% CI)	p-value
**Mean of grip strength measurements**								
**Baseline**	70	33.5 (10.7)	70	38.0 (12.1)				
**3 months**	68	35.0 (10.7)	64	38.7 (12.0)	-0.2 (-1.7, 1.3)	0.79	-0.2 (-1.7, 1.3)	0.82
**6 months**	64	34.9 (11.2)	65	38.5 (12.3)	0.2 (-1.3, 1.7)	0.78	0.2 (-1.3, 1.7)	0.75
**9 months**	64	34.4 (11.0)	66	38.4 (11.6)	-0.1 (-1.6, 1.5)	0.93	0.0 (-1.5, 1.5)	0.96
**12 months**	63	34.5 (10.7)	64	38.3 (11.9)	0.0 (-1.5, 1.5)	>0.99	0.0 (-1.5, 1.5)	0.97
**18 months**	62	34.8 (11.6)	55	38.4 (12.0)	0.2 (-1.4, 1.8)	0.8	0.2 (-1.3, 1.8)	0.77
**Overall active extension deficit of joint affected by treated nodule**								
**Baseline**	70	-6.7 (15.9)	70	-3.9 (18.3)				
**3 months**	68	-5.6 (17.7)	66	-2.7 (20.3)	-0.1 (-2.9, 2.6)	0.92	-0.1 (-2.9, 2.7)	0.95
**6 months**	64	-4.7 (17.8)	65	-1.7 (22.0)	0.0 (-2.8, 2.8)	>0.99	0.1 (-2.7, 2.9)	0.96
**9 months**	64	-4.3 (18.6)	66	0.3 (23.1)	-0.5 (-3.4, 2.3)	0.71	-0.5 (-3.3, 2.3)	0.75
**12 months**	63	-2.3 (20.4)	65	0.3 (25.1)	1.0 (-1.8, 3.9)	0.47	1.1 (-1.7, 3.9)	0.44
**18 months**	62	-0.4 (24.0)	55	-3.4 (23.1)	2.0 (-0.9, 4.9)	0.17	2.1 (-0.8, 5.0)	0.15
**MCP: active extension deficit of joint affected by treated nodule**								
**Baseline**	54	-13.0 (10.9)	60	-8.2 (15.8)				
**3 months**	52	-12.5 (12.7)	58	-7.1 (17.1)	0.2 (-2.6, 3.1)	0.87	0.4 (-2.5, 3.2)	0.79
**6 months**	50	-10.6 (13.9)	57	-6.4 (18.6)	0.8 (-2.0, 3.7)	0.57	1.0 (-1.9, 3.8)	0.51
**9 months**	50	-11.0 (12.6)	58	-4.8 (19.2)	0.1 (-2.8, 2.9)	0.95	0.2 (-2.6, 3.1)	0.87
**12 months**	49	-9.6 (14.4)	57	-5.4 (20.4)	1.7 (-1.1, 4.6)	0.24	1.8 (-1.0, 4.7)	0.21
**18 months**	49	-8.4 (17.0)	51	-6.5 (19.9)	1.6 (-1.3, 4.5)	0.27	1.8 (-1.1, 4.6)	0.23

	Adalimumab (n=70)		Saline (n=70)		Unadjusted treatment effect*		Adjusted treatment effect*	
	**n**	**Mean (SD)[1]**	**n**	**Mean (SD)[1]**	**Difference (95% CI)**	**p-value**	**Difference (95% CI)**	**p-value**
**PIP: active extension deficit of joint affected by treated nodule**								
**Baseline**	16	14.6 (11.0)	10	22.3 (6.7)				
**3 months**	16	16.6 (12.4)	8	29.1 (10.8)	-0.8 (-10.5, 8.9)	0.87	-3.1 (-12.2, 5.9)	0.50
**6 months**	14	16.4 (13.9)	8	32.4 (11.6)	-3.3 (-13.3, 6.6)	0.51	-5.2 (-14.4, 4.0)	0.27
**9 months**	14	19.7 (17.0)	8	37.0 (14.7)	-4.7 (-14.6, 5.2)	0.35	-6.5 (-15.7, 2.6)	0.16
**12 months**	14	23.2 (17.8)	8	40.5 (17.9)	-4.7 (-14.6, 5.2)	0.35	-6.5 (-15.7, 2.6)	0.16
**18 months**	13	29.9 (22.6)	4	36.0 (27.7)	-1.3 (-12.4, 9.9)	0.83	-3.0 (-13.5, 7.6)	0.58
**Overall passive extension deficit of joint affected by treated nodule**								
**Baseline**	69	2.8 (6.5)	70	2.9 (7.6)				
**3 months**	68	2.4 (6.2)	66	3.2 (7.8)	-0.6 (-3.0, 1.8)	0.62	-0.7 (-3.0, 1.7)	0.57
**6 months**	64	2.6 (7.6)	65	4.6 (9.8)	-1.3 (-3.7, 1.1)	0.29	-1.3 (-3.7, 1.0)	0.27
**9 months**	64	2.9 (8.3)	66	5.0 (11.7)	-1.4 (-3.8, 1.0)	0.26	-1.4 (-3.8, 0.9)	0.24
**12 months**	63	3.3 (10.2)	65	5.0 (12.8)	-1.4 (-3.8, 1.0)	0.26	-1.4 (-3.8, 1.0)	0.25
**18 months**	62	5.3 (13.5)	55	2.6 (10.5)	1.0 (-1.5, 3.4)	0.44	0.9 (-1.5, 3.4)	0.45
**MCP: passive extension deficit of joint affected by treated nodule**								
**Baseline**	53	0.3 (2.1)	60	1.6 (6.1)				
**3 months**	52	0.0 (0.0)	58	1.4 (5.3)	-0.7 (-2.4, 0.9)	0.37	-0.7 (-2.4, 0.9)	0.37
**6 months**	50	0.3 (2.0)	57	2.5 (7.6)	-1.5 (-3.2, 0.1)	0.065	-1.5 (-3.2, 0.1)	0.064
**9 months**	50	0.0 (0.0)	58	1.9 (7.0)	-1.1 (-2.7, 0.5)	0.18	-1.1 (-2.7, 0.5)	0.18
**12 months**	49	0.0 (0.0)	57	1.4 (5.7)	-1.1 (-2.7, 0.5)	0.19	-1.1 (-2.7, 0.5)	0.19
**18 months**	49	0.7 (4.6)	51	0.5 (3.4)	-0.1 (-1.7, 1.6)	0.95	-0.1 (-1.7, 1.6)	0.94

	Adalimumab (n=70)		Saline (n=70)		Unadjusted treatment effect*		Adjusted treatment effect*	
	**n**	**Mean (SD)[1]**	**n**	**Mean (SD)[1]**	**Difference (95% CI)**	**p-value**	**Difference (95% CI)**	**p-value**
**PIP: passive extension deficit of joint affected by treated nodule**								
**Baseline**	16	11.0 (9.2)	10	11.0 (10.3)				
**3 months**	16	10.0 (9.6)	8	16.0 (11.0)	-2.8 (-11.9, 6.2)	0.54	-3.9 (-11.7, 3.9)	0.32
**6 months**	14	11.1 (12.8)	8	19.8 (10.3)	-4.5 (-13.7, 4.7)	0.33	-4.8 (-12.8, 3.1)	0.23
**9 months**	14	13.1 (13.8)	8	27.3 (15.1)	-10.0 (-19.2, -0.8)	0.034	-10.2 (-18.2, -2.3)	0.012
**12 months**	14	14.7 (17.7)	8	30.0 (20.4)	-11.1 (-20.3, -1.9)	0.018	-11.4 (-19.4, -3.5)	0.0049
**18 months**	13	22.7 (20.7)	4	30.0 (26.8)	-9.1 (-20.0, 1.8)	0.10	-9.1 (-19.0, 0.7)	0.069

Metacarpophalangeal (MCP)and proximal interphalangeal (PIP) joint data are shown based on independent blinded assessment of baseline photographs and hand diagrams of whether the treated nodule affected the MCP and PIP, respectively. Due to low numbers for the PIP joint, results should be interpreted with caution.
Missing baseline data were mean imputed in the analysis model.
**adjusted differences: adjusted for baseline scores, site, age; unadjusted differences: only adjusted for baseline scores*
[1]Observed data are presented without imputation for missing data

Supplemental Figure S5: Pain during and immediately after injection

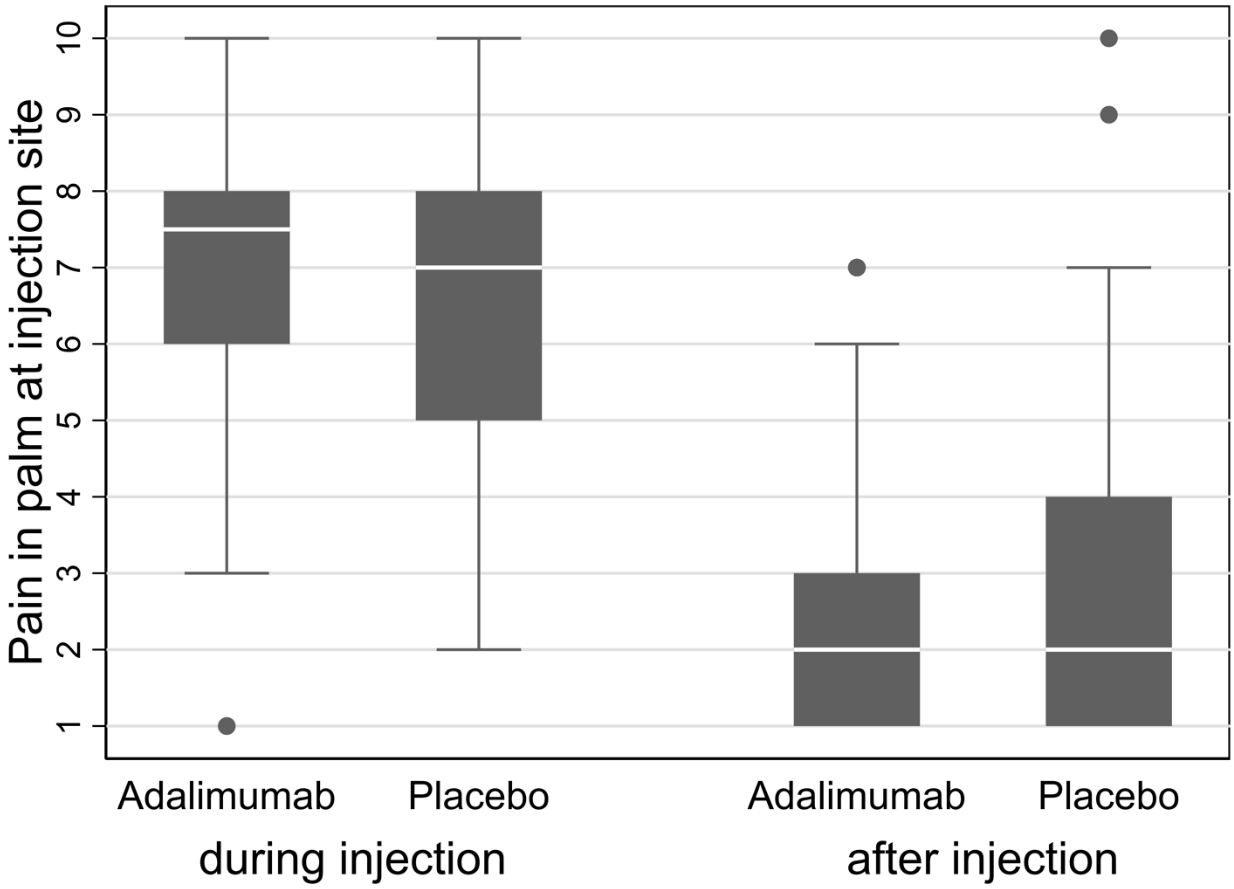

Pain was self-reported and measured on a 1-10 scale, with higher values indicating higher levels of pain.

Supplemental Figure S6: Circulating levels of adalimumab

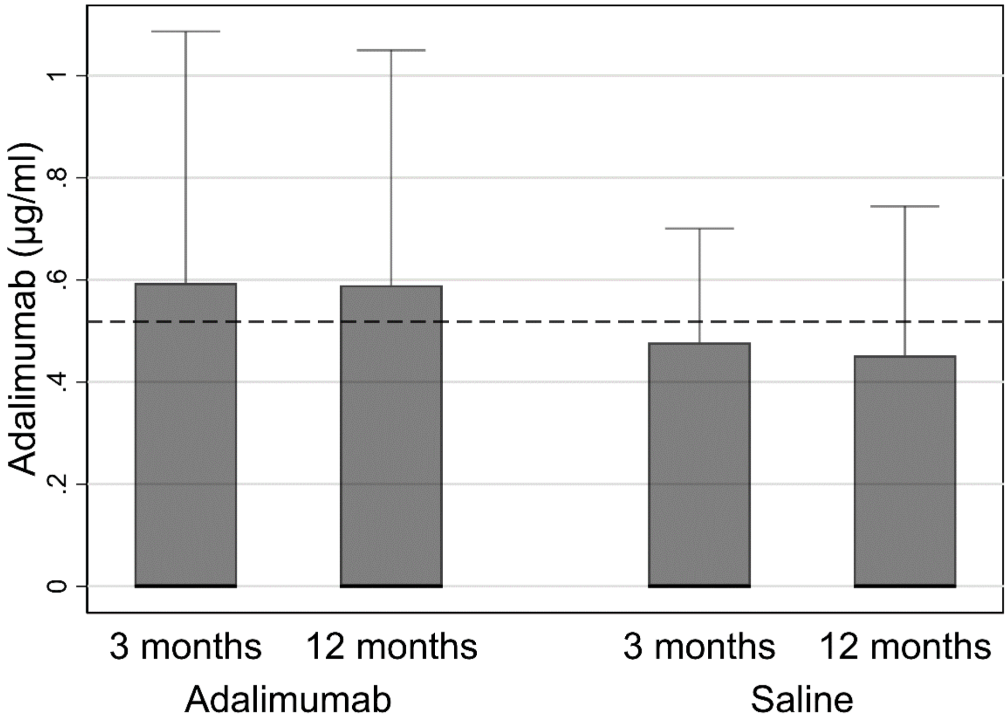

The median levels (0 for all groups) are indicated by the black lines. The limit of detection (0.518 µg/ml) is indicated by the dashed line.

Supplemental Figure S7: Quantitative assay for antibodies to adalimumab antibodies

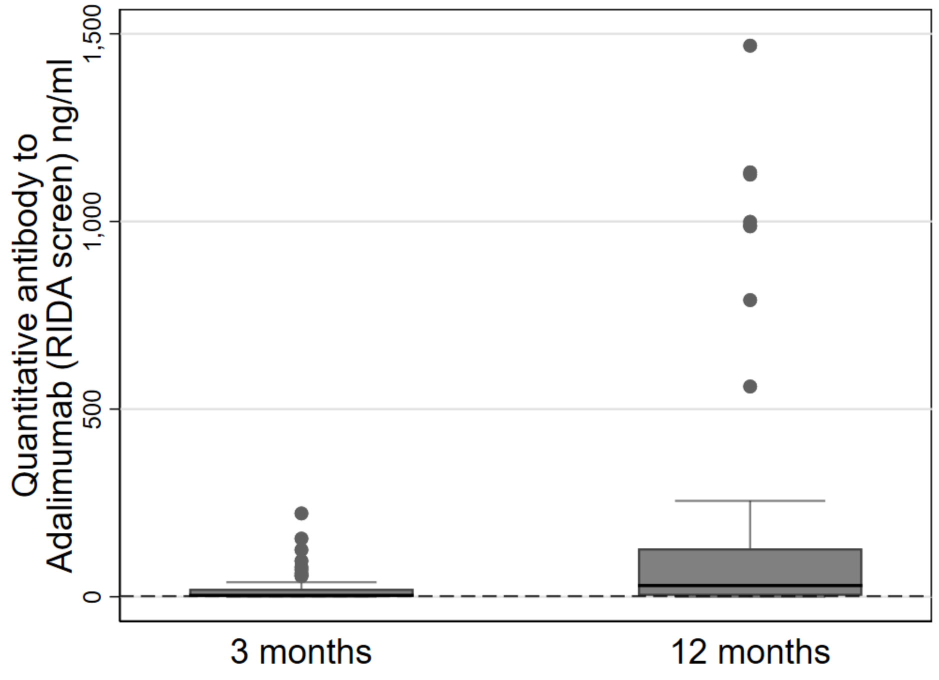

Figure 4a: Quantitative assay for antibodies to adalimumab in the adalimumab arm

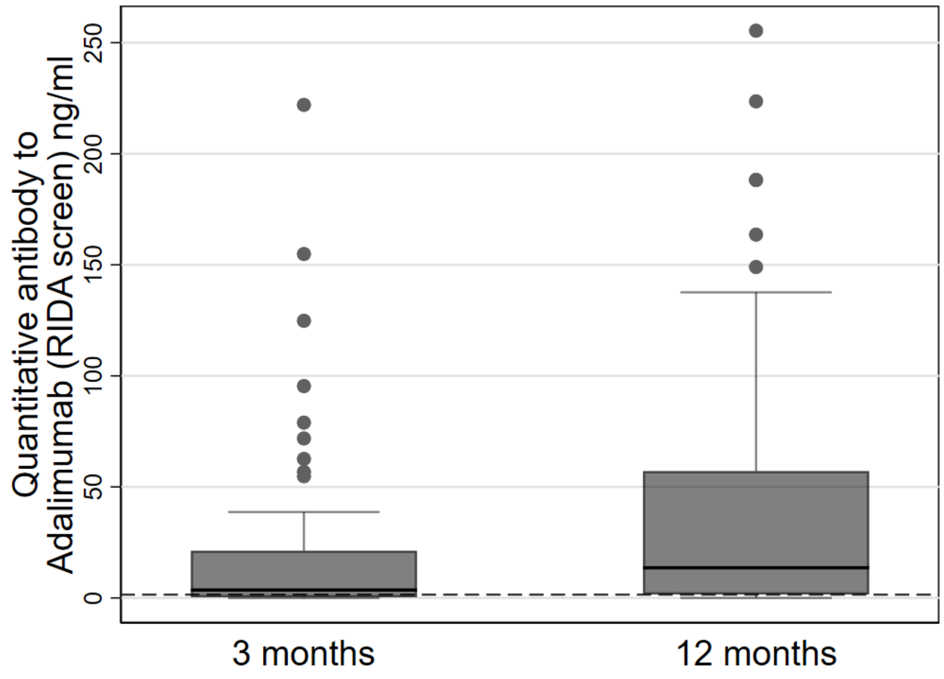

Figure 4b: Quantitative assay for antibodies to adalimumab in the adalimumab arm with values > 500ng/ml removed

The median levels are indicated by the solid black lines. The limit of detection (1.5 ng/ml) is indicated by the dashed line.

Supplemental Figure S8: Relationship between the concentration of circulating antibodies to adalimumab (quantitative RIDA screen) and change in durometer readings, nodule area, feret or height

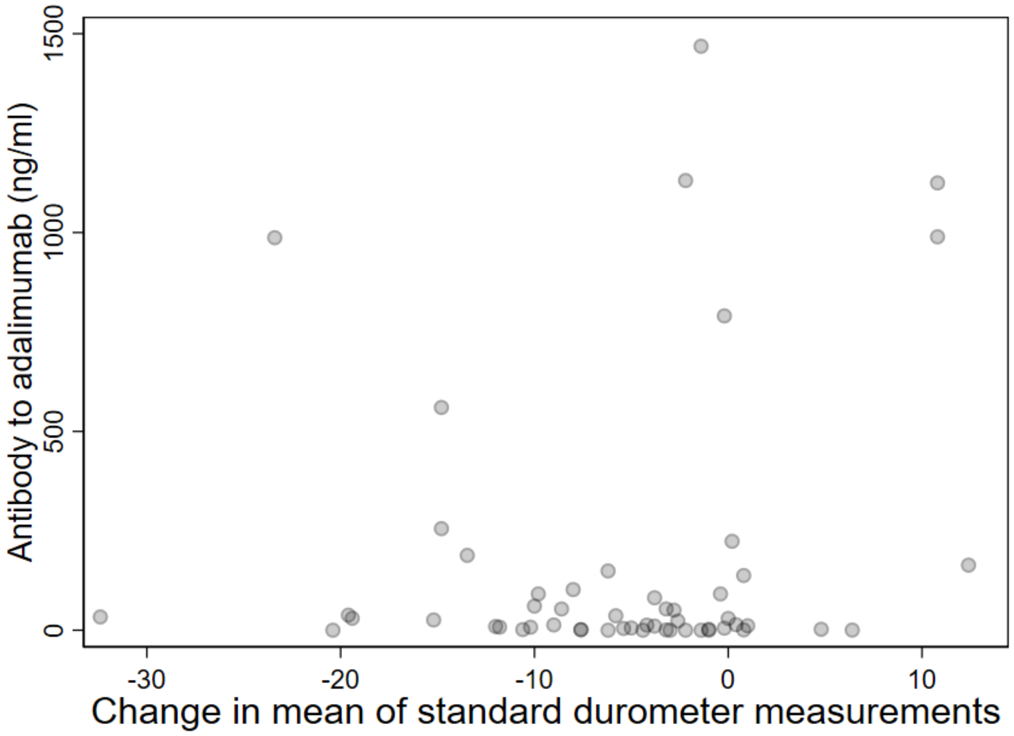

Figure 5a: Scatter plot of concentration of circulating antibodies to adalimumab at 12 months vs. change in nodule hardness [standard durometer (baseline to 12 months)]

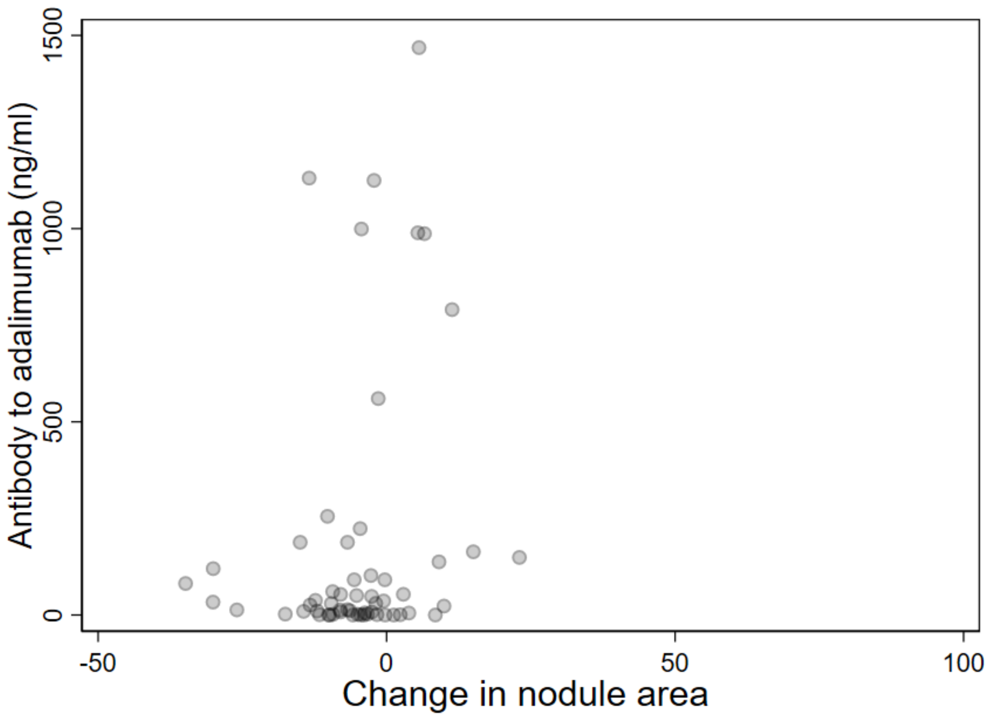

Figure 5b: Scatter plot of concentration of circulating antibodies to adalimumab at 12 months vs. change in nodule area (mm^2) (baseline to 12 months)

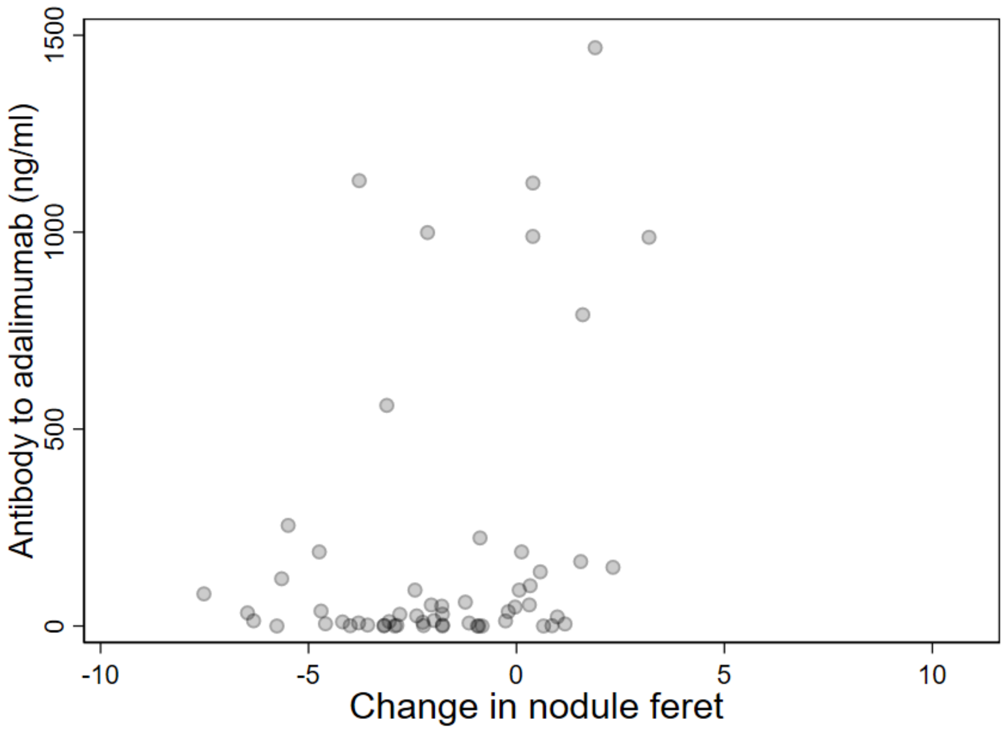

Figure 5c: Scatter plot of concentration of circulating antibodies to adalimumab at 12 months vs. nodule feret (mm^2) at 12 months

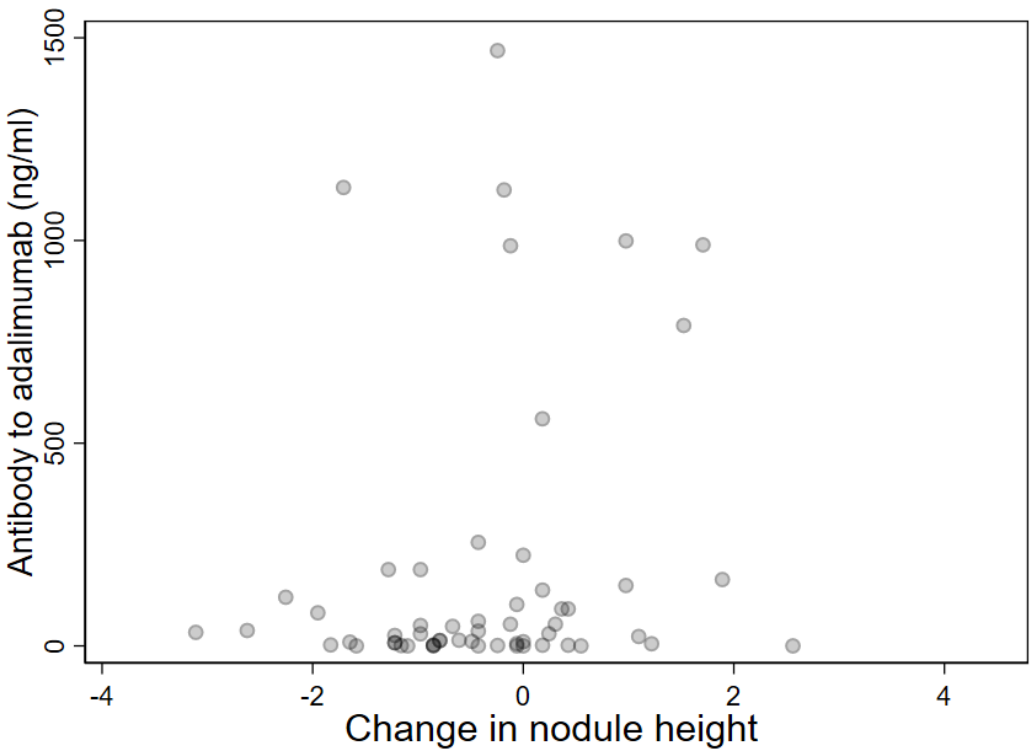

Figure 5d: Scatter plot of concentration of circulating antibody to adalimumab at 12 months vs. change in nodule height (mm) (baseline to 12 months)

References:

1. Brazier JE, Yang Y, Tsuchiya A, Rowen DL. A review of studies mapping (or cross walking) non-preference based measures of health to generic preference-based measures. *Eur J Health Econ* 2010; **11**(2): 215-25.

2. Longworth L, Rowen D. NICE DSU technical support document 10: the use of mapping methods to estimate health states utility values. *NICE Decision Support Unit Technical Suport* 2011; http://nicedsu.org.uk/technical-support-documents/

3. Hindocha S, Stanley JK, Watson S, Bayat A. Dupuytren's diathesis revisited: Evaluation of prognostic indicators for risk of disease recurrence. *J Hand Surg* 2006; **31**(10): 1626-34.

4. Bland JM, Altman DG. Measuring agreement in method comparison studies. *Stat Methods Med Res* 1999; **8**(2): 135-60.
